# CREB-B acts as a key mediator of NPF/NO pathway involved in phase-related locomotor plasticity in locusts

**DOI:** 10.1371/journal.pgen.1008176

**Published:** 2019-05-31

**Authors:** Li Hou, Beibei Li, Ding Ding, Le Kang, Xianhui Wang

**Affiliations:** 1 State Key Laboratory of Integrated Management of Pest Insects and Rodents, Institute of Zoology, Chinese Academy of Sciences, Beijing, China; 2 Beijing Institutes of Life Science, Chinese Academy of Sciences, Beijing, China; Katholieke Universiteit Leuven, BELGIUM

## Abstract

Gene expression changes in neural systems are essential for environment-induced behavioral plasticity in animals; however, neuronal signaling pathways mediating the effect of external stimuli on transcriptional changes are largely unknown. Recently, we have demonstrated that the neuropeptide F (NPF)/nitric oxide (NO) signaling pathway plays a regulatory role in phase-related locomotor plasticity in the migratory locust, *Locusta migratoria*. Here, we report that a conserved transcription factor, cAMP response element-binding protein B (CREB-B), is a key mediator involved in the signaling pathway from NPF2 to NOS in the migratory locust, triggering locomotor activity shift between solitarious and gregarious phases. We find that CREB-B directly activates brain *NOS* expression by interacting with *NOS* promoter region. The phosphorylation at serine 110 site of CREB-B dynamically changes in response to population density variation and is negatively controlled by NPF2. The involvement of CREB-B in NPF2-regulated locomotor plasticity is further validated by RNAi experiment and behavioral assay. Furthermore, we reveal that protein kinase A mediates the regulatory effects of NPF2 on CREB-B phosphorylation and *NOS* transcription. These findings highlight a precise signal cascade underlying environment-induced behavioral plasticity.

## Author summary

The migratory locust, *Locusta migratoria*, is a worldwide agricultural pest that displays a remarkable density-dependent phase polyphenism, where being kept in a crowd triggers individuals to transit from the sedentary solitarious phase into the high-active gregarious phase. So, the migratory locust has been regarded as an excellent study model for environment-induced behavioral plasticity. Our previous finding shows that NPF2-regulated *NOS* transcription plays important roles in phase-related locomotor plasticity in the locust. Here, we further demonstrate that phosphorylated CREB-B directly activates *NOS* transcription in the pars intercerebralis, thus mediates phase-related locomotor plasticity. Further studies show that the levels of CREB-B phosphorylation is positively correlated with the crowding treatment and suppressed by NPF2. Among several candidate kinases, protein kinase A is demonstrated to transmit the inhibitory effects of NPF2 on CREB-B phosphorylation and *NOS* transcription. Our study provides deep insight into the precise regulatory mechanisms underlying environment-induced behavioral plasticity.

## Introduction

Animals can adjust to a changing environment by developing alternative behavioral phenotypes that improve their fitness; this phenomenon is known as “behavioral plasticity” [1–3]. Environmental stimuli acts directly on the nervous system and induces short-term changes in neural and endocrine activity, or long-term changes in gene expression, thus lead to behavioral alterations at different time scales [4, 5]. Various neural modulators with distinct profiles of molecular action are involved in this process [6–8]. In a given context, long-term behavioral plasticity is greatly shaped by transcriptional changes in key genes that are governed intricately by the interactions of neural modulators in the brain [9–11].

Transcription factors (TFs) play central roles in the regulation of behavioral plasticity through integrating neural signals and downstream transcriptional events [8, 12, 13]. Nuclear TFs primarily modify behavioral performance by binding to the regulatory region (e.g., promoter or enhancer) of their target genes [14, 15]. With distinct stimulation, TFs undergo either expression alteration or protein modification changes that affect their subcellular location, binding activity, or stability; and then result in transcriptional changes of downstream behavior-related genes [16–18]. For example, Fos family proteins can be induced rapidly or transiently in specific regions (such as nucleus accumbens and dorsal striatum) by drug abuse, thus influencing rewarding and locomotor behaviors [19]. Nuclear factor-κB (NF-κB) family members are essential for hippocampus-dependent long-term memory formation by regulating memory-associated genes [20]. Therefore, uncovering the precise signaling cascade by which TFs respond to upstream signal and regulate downstream gene transcription will provide insights into the regulatory mechanism underlying environment-induced behavioral plasticity.

The migratory locust, *Locusta migratoria*, displays phase-related behavioral plasticity in response to population density variation [21, 22]. Gregarious locusts are highly active and attracted to their conspecifics, whereas solitarious locusts are cryptic and repelled from other individuals. The attraction index and locomotion activity substantially decrease during the isolation of gregarious locusts but are promoted by forced crowding in solitarious locusts [23]. During the time-course processes of solitarization and gregarization, gene expression profiles in the locus brain display dynamic changes [24]. The regulatory roles of several key genes have been revealed in behavioral phase transition [23, 25]. In particular, we have recently demonstrated that a neural neuropeptide F (NPF)/nitric oxide (NO) signaling pathway plays an essential role in phase-related locomotor plasticity, which results from the sequential changes in the phosphorylation and transcriptional states of nitric oxide synthase (NOS) as regulated by NPF1a and NPF2 systems, respectively [26]. NPF1a-regulated NOS phosphorylation initiates an immediate change in locomotor activity, whereas NPF2-regulated *NOS* transcription is responsible for long-term locomotor plasticity. However, the mechanism underlying *NOS* transcription changes as regulated by the up-stream TFs in response to population density variation is still unknown.

In this study, we aim to identify the TF activating *NOS* transcription in response to population density change. Our results uncovered several novel components of the NPF/NO signaling cascade underlying phase-related locomotor plasticity in locusts.

## Results

### CREB-B directly up-regulates *NOS* transcription in the locust brain

Our previous work showed that the mRNA level of *NOS* robustly responded to the changes in population density [26]. To further uncover the regulatory mechanisms of *NOS* transcription, we first identified a ~1.5 kb genomic region located upstream of *NOS* coding sequence by genome walking based on the whole genome sequence of the migratory locust. The transcriptional activity of DNA constructs carrying the ~1.5 kb upstream region of *NOS* fused with luciferase gene cassette was stronger than that of the empty pGL4.1 vector (NC) in HEK293T cells (>10 fold, S1 Fig). By progressively truncating the upstream genome sequence of *NOS*, we found that a genomic region from -150 bp to -121 bp upstream of the ATG start codon may serve as the core promoter sequence of *NOS* (S1 Fig).

**Fig 1:**
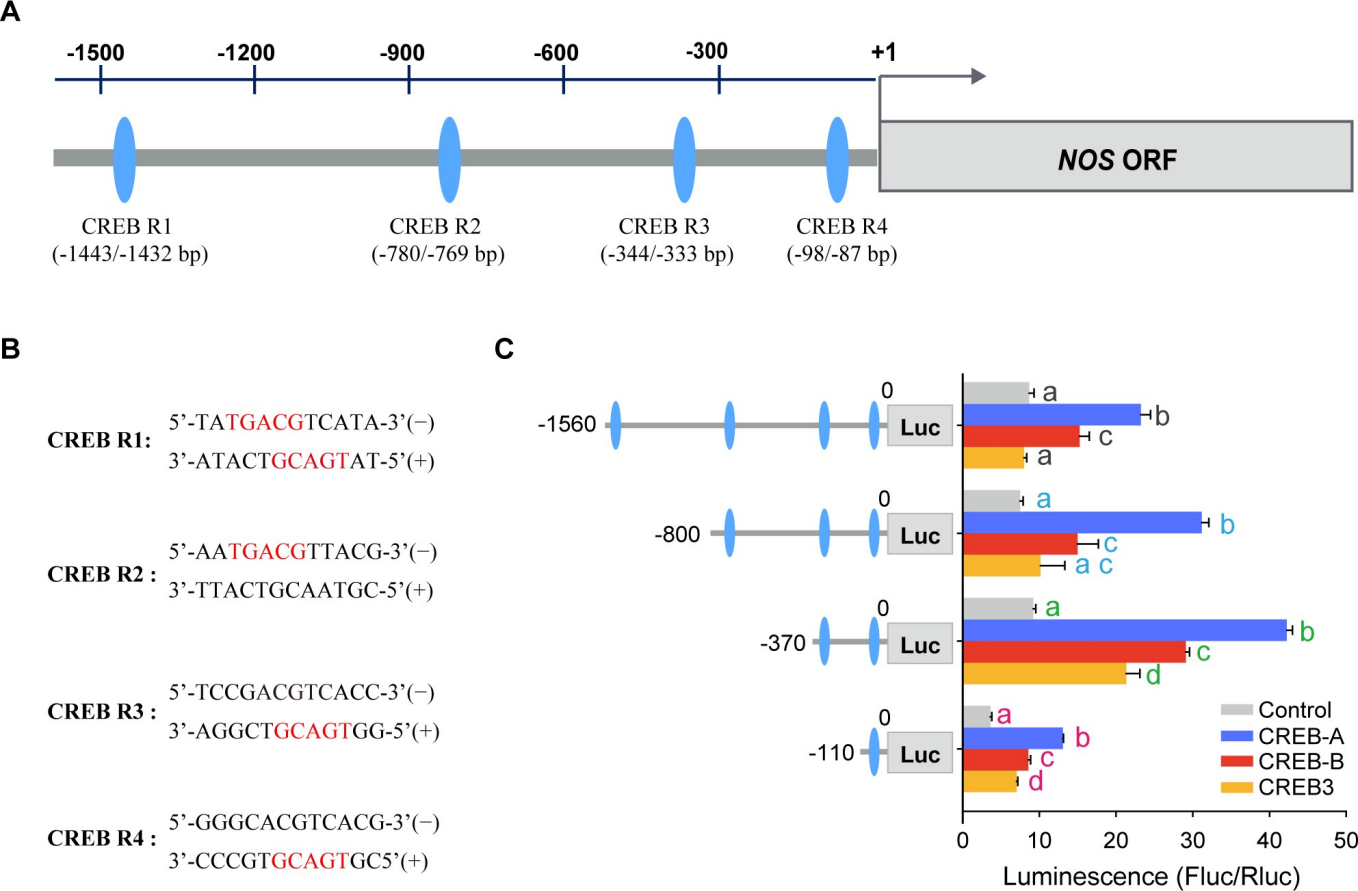
Transcriptional activity of CREBs for the upstream element of *NOS* in HEK293T cells. (A) Schematic representation of the location of CREB-response elements in the *NOS* promoter region. The grey bar and box indicate the promoter and coding region, respectively. The blue ellipses indicate CREB-response elements. Numbers indicate the distance from the translation initiation site (+1) of *NOS*. (B) Sequences of the four predicted CREB-response elements. Red letters show the core nucleotides of CREB-binding motif. (C) Effects of CREB over-expression on the progressive deletions of *NOS* promoter examined by dual-luciferase report system (n = 4 replicates). Data are indicated by mean±s.e.m., one-way ANOVA, *P* < 0.05. Different letters in same color indicate significant.

To identify candidate TFs responsible for *NOS* transactivation, we predicted *cis*-response elements (CREs) in the promoter/enhancer sequence of *NOS* gene by using two different software, MatInspector program and TANSFAC program [27, 28]. Only CREB CREs were confirmed in the regulatory region of *NOS* gene according to the prediction of two independent programs (Fig 1A). In the locust genome sequence, we identified three CREB family members named as CREB-A, CREB-B, and CREB3 according to their phylogenetic relationship (S2 Fig). Extremely low sequence identity was found among these three CREB proteins (13.55%, S3 Fig). We examined which one of these CREBs can enhance the transcriptional activity of *NOS* gene by using dual-luciferase reporter assay. Over-expression of CREB-A and CREB-B protein (fused with flag tag) can strongly increase the transcriptional activity of *NOS* promoter in HEK293T cells, even after the promoter region of *NOS* was reduced to -110 bp and contains only the fourth CREB CRE (CREB R4, -98 to -87 bp upstream of NOS translational start site, Fig 1C and S4 Fig). Whereas over-expression of CREB3 only enhanced the transcriptional activity of DNA constructs containing CREB R3 and CREB R4 in HEK293T cells.

**Fig 2:**
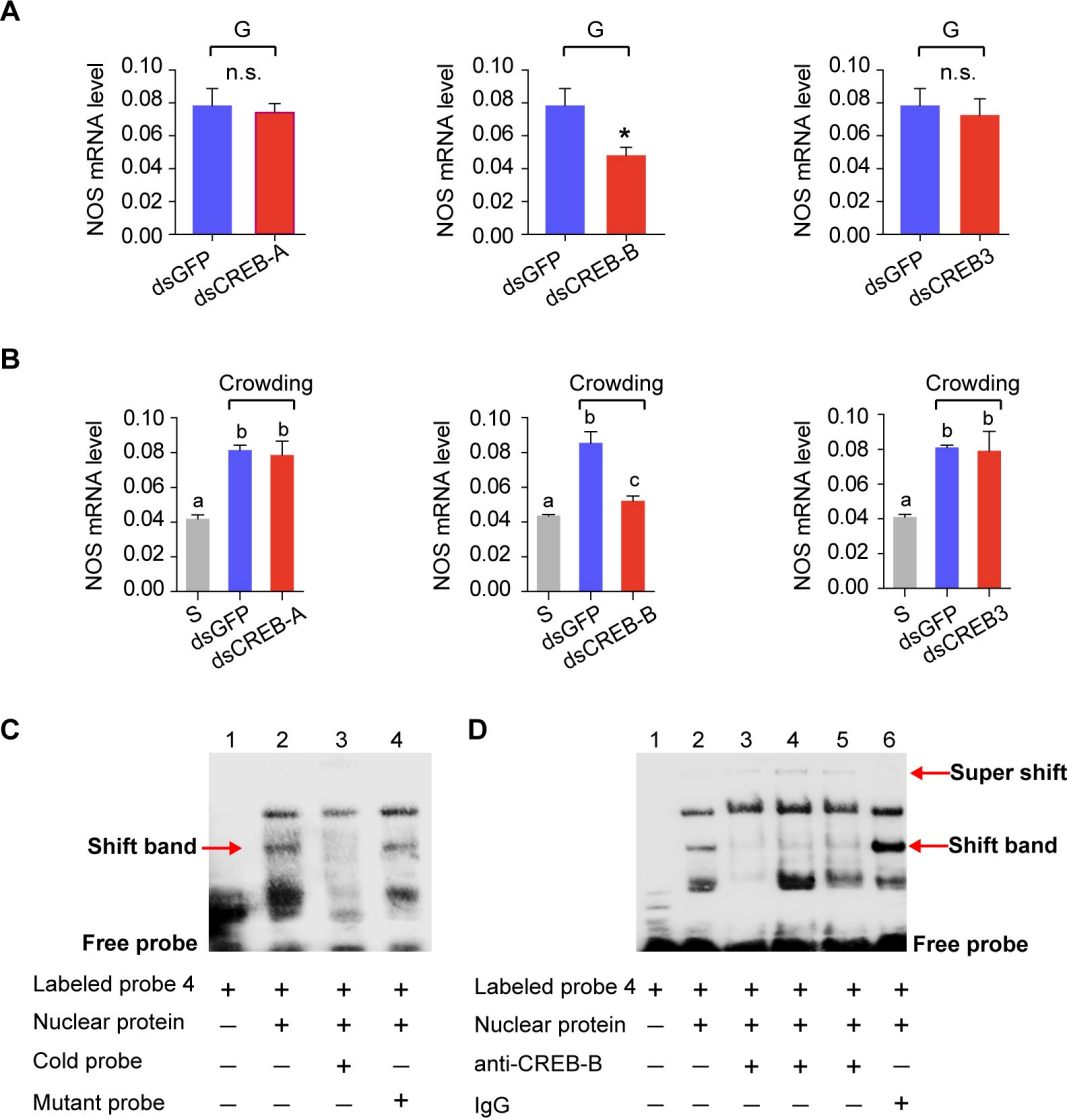
CREB-B interacts with NOS promoter region and regulates *NOS* transcription in the locust brain. Effects of RNAi-mediated silencing of CREB encoding genes on *NOS* transcription (A) in the G-phase locusts (n = 4 replicates, 6–8 locusts/replicate, Student’s t-test, *P < 0.05) and (B) in the forced crowding S-phase locusts (n = 4 replicates, 6–8 locusts/replicate, one-way ANOVA, *P* < 0.05). (C) Electrophoretic mobility shift assay (EMSA) of binding of the nuclear proteins extracted from the brain tissues to the CREB-response element (CREB R4) of the *NOS* promoter. Nuclear proteins extracted from the locust brain were used to bind with the biotin-labeled CREB R4 probe (CTAGCGCGCGTGACGTGCCCCGGCT). The cold CREB R4 probe was unlabeled. For the mutated probe, TGACG at 11–15 nt of the wild type CREB R4 probe was replaced by TTTTT (CTAGCGCGCGTTTTTTGCCCCGGCT). (D) Validation of the binding of CREB-B protein with the *NOS* promoter by super-shift assay. Specific antibody of CREB-B (lane 3: monoclonal rabbit antibody against p(S133)-CREB1; lane 4: polyclonal antibody rabbit against p(S133)-CREB1; lane 5: polyclonal rabbit antibody against CREB-B) were used to form the supershift band. Rabbit IgG was used as the control.

The potential regulatory effects of these three CREBs on *NOS* expression *in vivo* were validated through RNA interference experiments. Only the knockdown of *CREB-B* significantly suppressed the *NOS* expression in the brains of gregarious locusts (Fig 2A and S5 Fig) and prevented the up-regulation of *NOS* transcription when solitary locusts were crowded (Fig 2B). However, the knockdowns of *CREB-A* or *CREB3* did not affect *NOS* transcription in either gregarious or solitary locusts (Fig 2A and 2B). These results suggested the CREB-B is required for *NOS* transcription in the locust brain.

We performed electrophoretic mobility shift assay (EMSA) to identify distinct CREB-B binding sites from the four putative CREB CREs (CREB R1, CREB R2, CREB R3, and CREB R4) in the *NOS* promoter region. Only CREB R4 probe could bind with the nuclear proteins isolated from brain tissues (Fig 2C and S6 Fig). The shift band disappeared after the cold probe (100 × over the labeled probe) was added (Fig 2C, lane 3) but was not affected by the mutant probe, in which five nucleotides were replaced (Fig 2C, lane 4). A super shift assay was further performed by using three different antibodies recognizing locust CREB-B (S7 Fig). The shift band receded, and a super-shifted band appeared after the CREB-B antibodies were added (Fig 2D, lane 3–5). By contrast, no super-shifted band was found in the control incubated with normal rabbit IgG (Fig 2D, lane 6), indicating that the nuclear protein binding CREB R4 should be CREB-B protein.

**Fig 3:**
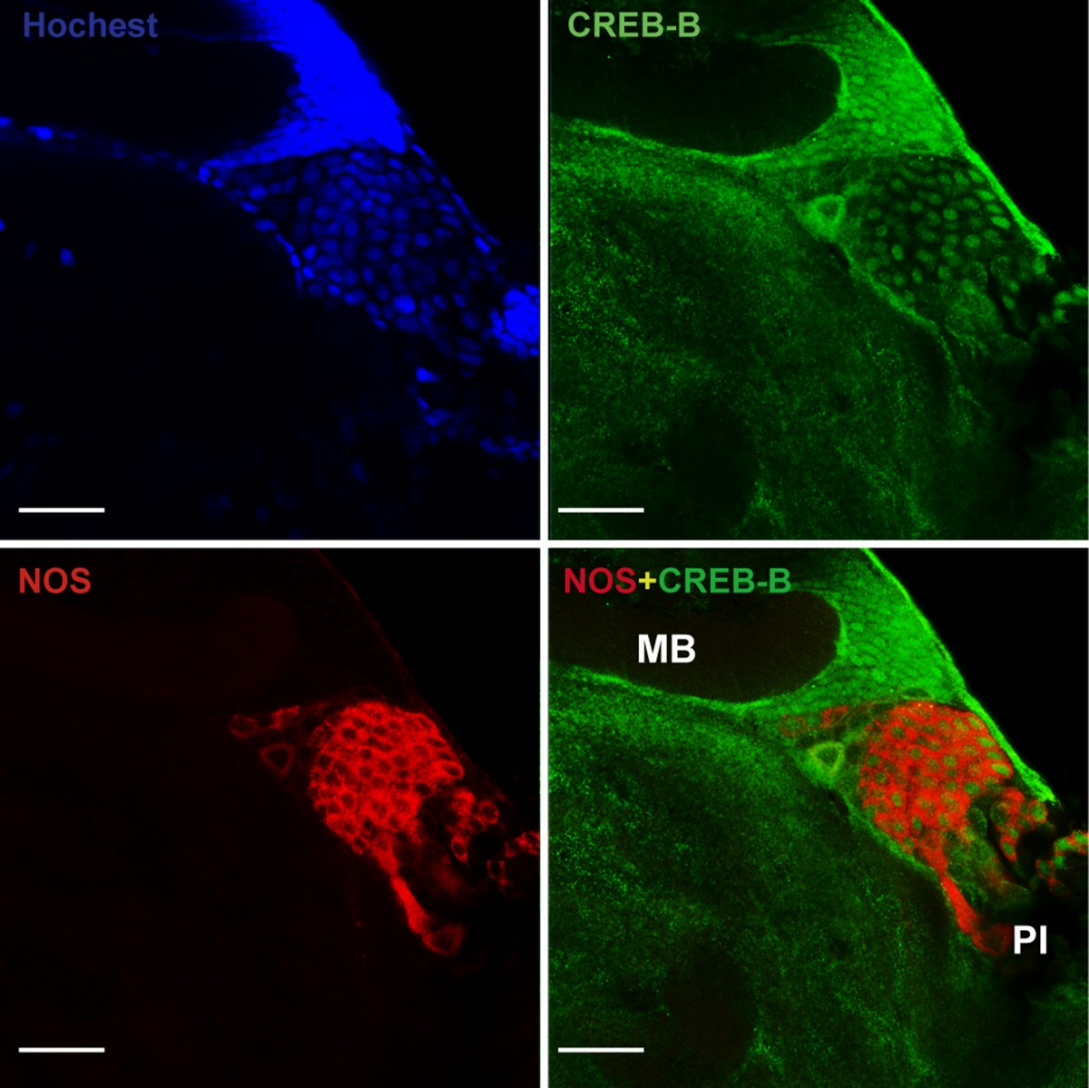
Subcellular localization of CREB-B and NOS in the pars intercerebralis (PI) of locust brain. Both CREB-B and NOS expressed in the neurons of PI. CREB-B mainly localized in the nuclei. Polyclonal antibody against p(S133)-CREB1 (1:100) and uNOS (1:100) were used in the immunohistochemistry assay. Green indicates CREB-B staining, red indicates NOS staining. Bar represents 100 μm.

Our previous study showed that NOS is extensively expressed in the neurons of the pars intercerebralis (PI) [26]. Here, by using double immunofluorescence staining, we found that CREB-B was also mainly localized in the nuclei of the neurons expressing NOS in PI (Fig 3 and S8 Fig). These results indicated that CREB-B may serve as a direct modulator of *NOS* expression in the locust brain.

### CREB-B is essential for phase-related locomotor plasticity

**Fig 4:**
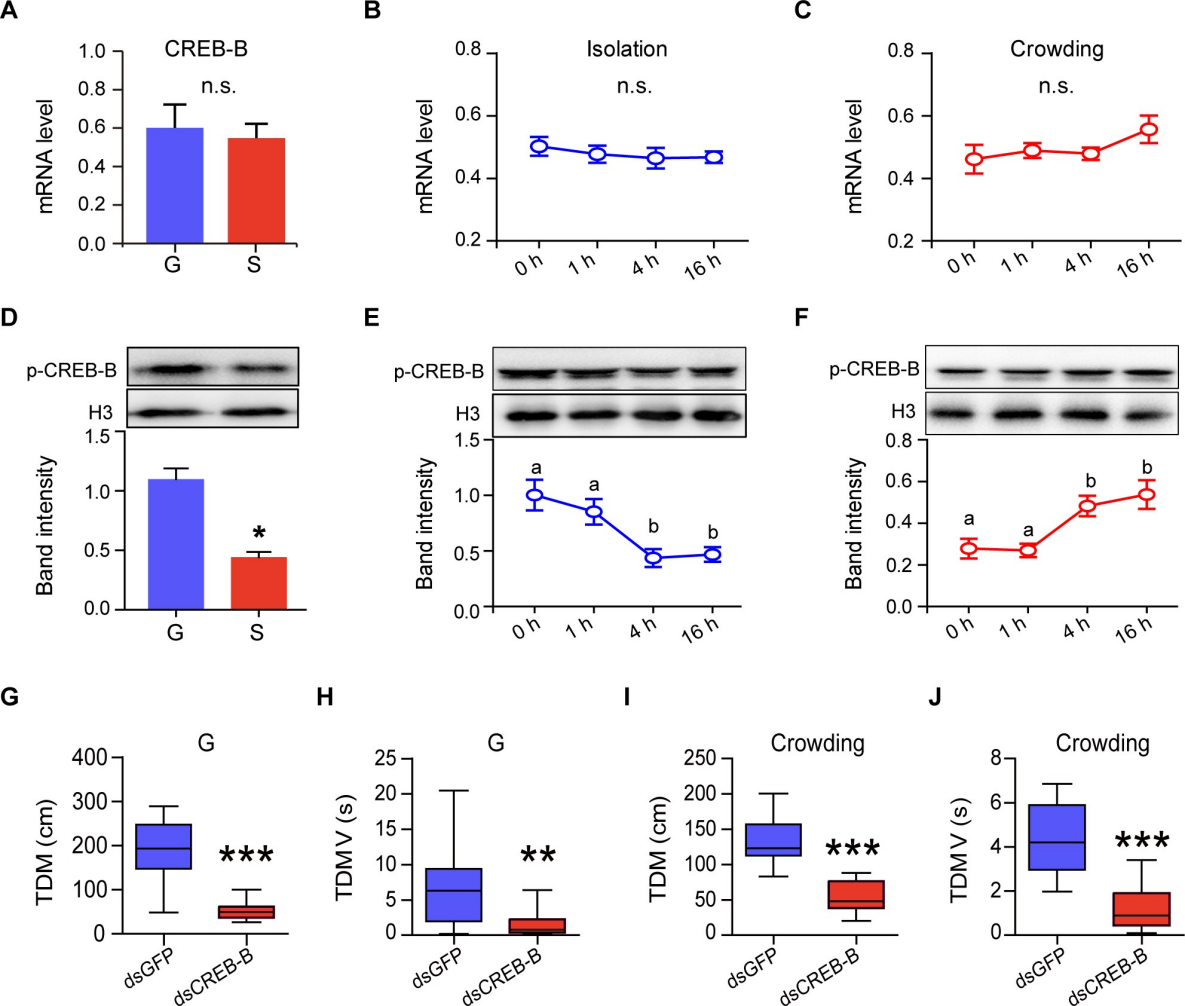
CREB-B displays dynamic phosphorylation levels and regulates locomotor activity during locust phase transition. (A) Total distance moved (TDM) and (B) total duration of movement (TDMV) in G-phase locusts after transcript knockdown of *CREB-B* (n ≥ 17 locusts, **P < 0.01, ***P < 0.001 with Student’s t-test). (C) TDM and (D) TDMV in crowded (32 h) S-phase locusts after transcript knockdown of *CREB-B* (n ≥ 15 locusts, ***P < 0.001 with Student’s t-test). (E) *CREB-B* transcription levels in G-phase and S-phase locust brains. Time course of *CREB-B* transcription level during (F) the isolation of G-phase locusts and (G) the crowding of S-phase locusts (n = 4 replicates, 6–8 locusts/replicate, n.s. indicates not significant). (H) The levels of phospho-CREB-B differ in G-phase and S-phase locust brains (n = 3 replicates, 8–12 locusts/replicate, *; *P* < 0.05 with Student’s t-test). Time course of phospho-CREB-B during (I) the isolation of G-phase locusts and (J) the crowding of S-phase locusts. Data are presented as mean ± s.e.m. Significant differences are denoted by letters (n = 3 replicates, 8–12 locusts/replicate, one-way ANOVA, *P* < 0.05).

To verify whether or not CREB-B is involved in the behavioral phase transition in the locust, we conducted behavioral assay after the knockdown of *CREB-B* gene in the locusts. The total distance moved (TDM) and total duration of movement (TDMV) were robustly reduced in the gregarious locusts injected with dsCREB-B (Fig 4A and 4B). Meanwhile, the knockdown of *CREB-B* also inhibited the enhancement of TDM and TDMV in the solitary locusts upon 32 h crowding, (Fig 4C and 4D). However, neither TDM nor TDMV was affected by *CREB-B* gene silencing in solitarious locusts (S9 Fig).

We found that there were no significant difference of the mRNA levels of *CREB-B* in brain tissues between gregarious and solitary locusts and between isolation and crowding (Fig 4E, 4F and 4G). A number of documents have suggested that CREB protein phosphorylation is a conserved and critical regulatory mechanism for transcriptional activation [29, 30]. Thus, we confirmed whether or not CREB-B could be phosphorylated in locust brain tissues. The phosphorylation site of human CREB1 protein and locust CREB-B share high identity in their phosphorylated kinase-inducible (KID) domains (S7A Fig). We found that only the antibody against serine133 of human CREB1 (corresponding to Ser110 of the locust CREB-B) can detect positive band with the predicted molecular weight of the locust CREB-B protein (S7B–S7D Fig). The phosphorylation of CREB-B (p-CREB-B) was further validated by RNAi experiments, in which the band intensity (detected by anti-p(S133-CREB1) was significant reduced after the knockdown of *CREB-B* gene (S7E Fig). Moreover, the level of p-CREB-B was higher in gregarious locust brains (Fig 4H) and dynamically changed during the time-course phase transition, in which p-CREB-B level remarkably decreased after 4 h isolation but increased after 4 h crowding (Fig 4I and 4J and S10 Fig). These results together with our previous findings suggested that CREB-B plays a regulatory role in phase-related locomotor activity.

### CREB-B participates in the regulation of NPF2 on locomotor activity during phase transition

**Fig 5:**
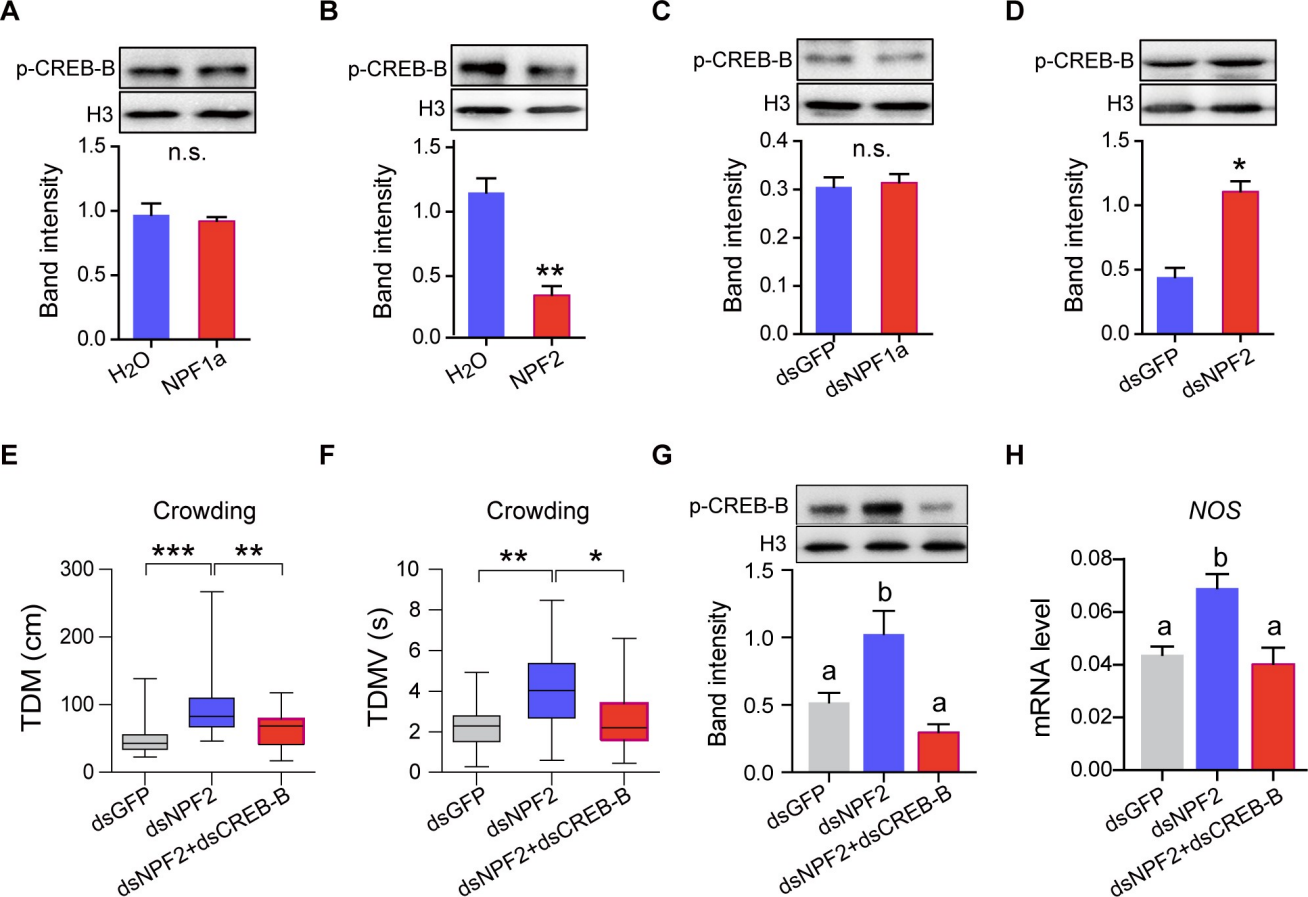
CREB-B modulates phase-related locomotor activity under NPF2 regulation. (A) and (B) Effects on p-CREB-B level after injection NPF1a or NPF2 peptide in G-phase locusts. (C) and (D) Effects on p-CREB-B level after transcript knockdown of *NPF1a* or *NPF2* in S-phase locusts (n = 4 replicates, 8–12 locusts/replicate, *P < 0.05, **P < 0.01 with Student’s t-test). (E) TDM and (F) TDMV in crowded (16 h) S-phase locusts after dual-knockdown of *NPF2* and *CREB-B* genes in crowded (16 h) S-phase locusts (n ≥ 20 locusts, * *P* < 0.05, **P < 0.01 with Student’s t-test). (G) p-CREB-B level and (H) *NOS* transcription level after dual-knockdown of *NPF2* and *CREB-B* genes in crowded S-phase locusts (n = 3 replicates, 6–8 locusts/replicate, one-way ANOVA, *P* < 0.05).

Considering that neuropeptide F, NPF2, modulates phase-related locomotor activity by suppressing *NOS* transcription [26], we determine whether or not CREB-B could mediate the regulatory effect of NPF2 on *NOS* transcription. We found that the p-CREB-B level strongly decreased after the injection of NPF2 peptide into gregarious locusts but was elevated after the knockdown of *NPF2* gene in solitary locusts (Fig 5B and 5D and S11 Fig). By contrast, the treatment with another neuropeptide F, NPF1a (either the full length or the truncated peptide) did not affect the levels of p-CREB-B (Fig 5A and 5C and S12 Fig). RNAi-mediated *CREB-B* knockdown completely blocked the up-regulation in both TDM and TDMV caused by *NPF2* knockdown upon the crowding treatment (Fig 5E and 5F). Furthermore, RNAi-mediated *CREB-B* knockdown also inhibited the increase of p-CREB-B levels and *NOS* transcription (Fig 5G and 5H), though the knockdown of *NPF2* did not affect *CREB-B* transcription (S13 Fig). These results revealed that CREB-B is an essential mediator of phase-related locomotion under the control of NPF2 signal at the phosphorylation level.

### Protein kinase A (PKA) mediates suppressive effects of NPF2 on CREB-B phosphorylation and *NOS* transcription

**Fig 6:**
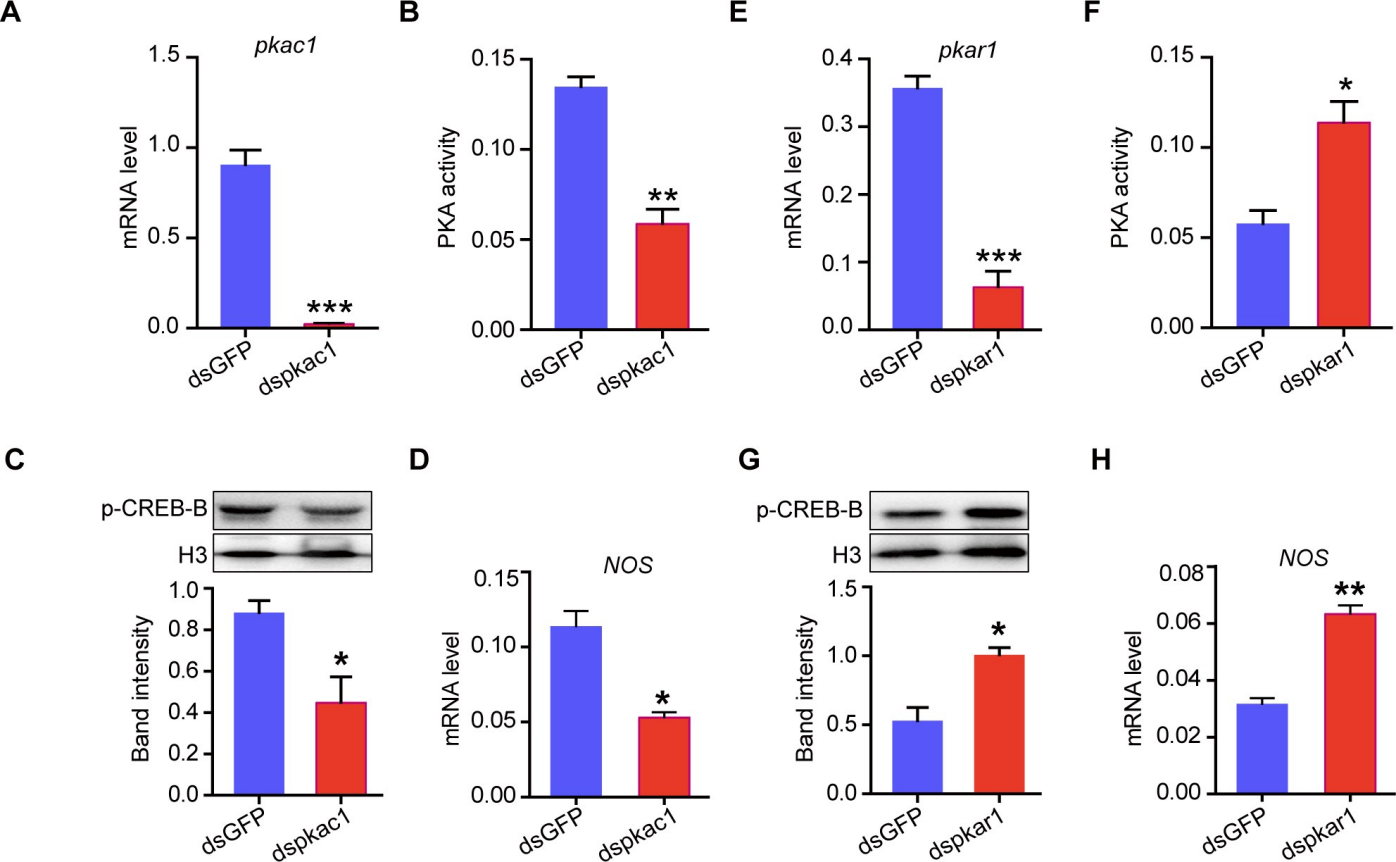
RNAi-mediated interferences of PKA activity change CREB-B phosphorylation and *NOS* transcription levels. (A) RNAi efficiency and (B) PKA activity after transcript knockdown of the catalytic C1 subunit of PKA encoding gene (*pkac1*). Effects on (C) p-CREB-B and (D) *NOS* transcription levels after the transcript knockdown of *pkac1* in G-phase locusts. (E) RNAi efficiency and (F) PKA activity after transcript knockdown of the regulatory R1 subunit of PKA encoding gene (*pkar1*). Effects on (G) p-CREB-B and (H) *NOS* transcription levels after the transcript knockdown of *pkar1* in S-phase locusts (n = 3 replicates, 6–8 locusts/replicate for qPCR, 8–12 locusts/replicate for western blot, *P < 0.05, **P < 0.01, ***P < 0.001 with Student’s t-test).

We predicted candidate kinases that may catalyze CREB-B phosphorylation by using NetPhos program (www.cbs.dtu.dk/services/NetPhos/). We found that the Ser110 site of CREB-B is the most potentially phosphorylated by three kinases, namely, PKC, PKA, and ribosomal protein S6 kinase (S6K) (S1 Table). By using RNAi-mediated knockdown, we further examined the effects of these three kinases and another three reported kinases on p-CREB-B and *NOS* expression [31, 32]. The levels of p-CREB-B and *NOS* expression were significantly decreased by RNAi-mediated interferences of PKA activity. Suppressing the PKA activity by the knockdown of its catalytic C1 subunit (*pkac1*) notably inhibited the p-CREB level and *NOS* transcription (Fig 6A–6D). By contrast, enhancing the PKA activity by the knockdown of its regulatory R1 subunit (*pkar1*) induced opposite effects (Fig 6E–6H). The silencing of other five kinase encoding genes did not significantly change the levels of p-CREB and *NOS* transcription (S14 Fig). These results indicated that PKA is the key main regulator of p-CREB-B in the locust brain.

**Fig 7:**
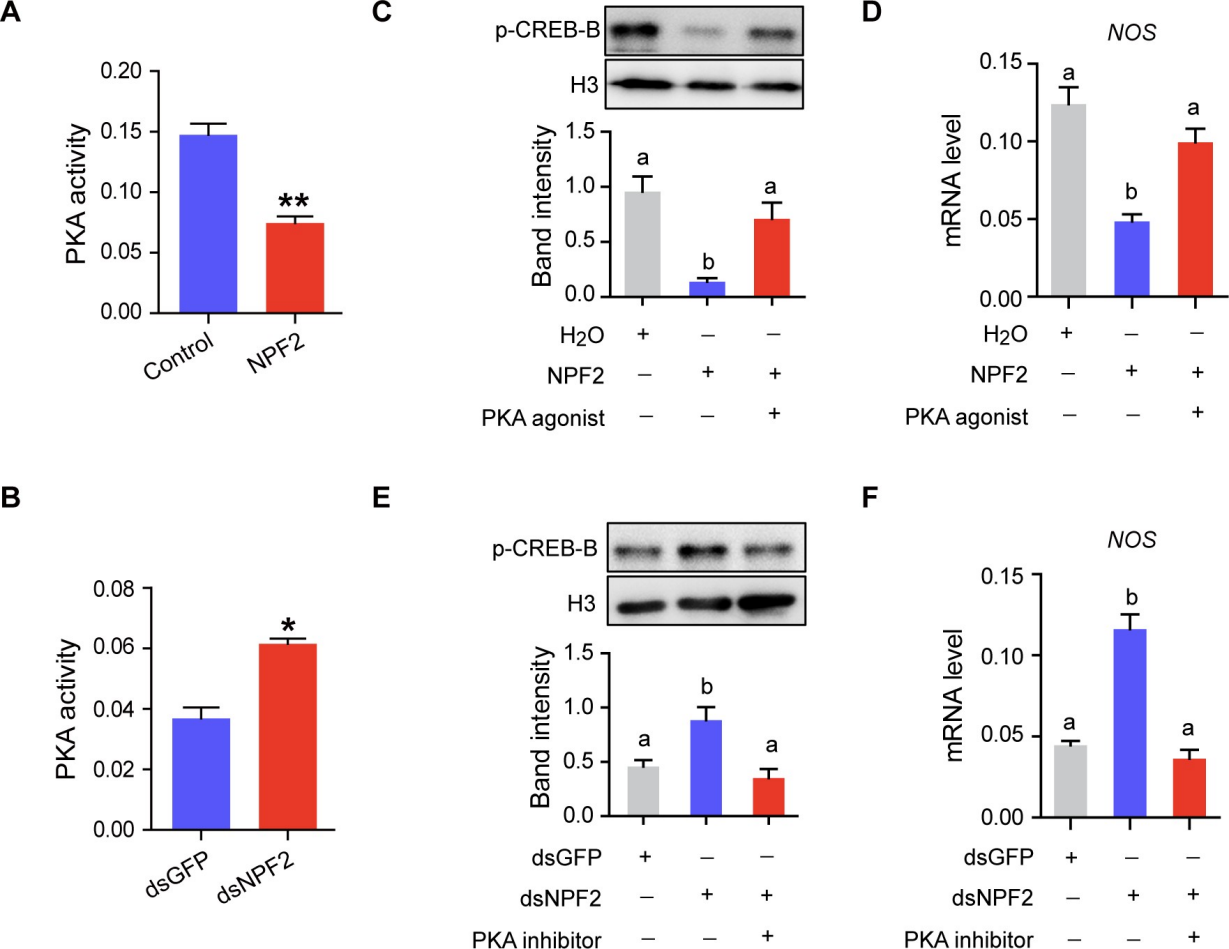
PKA mediates the regulatory effects of NPF2 on CREB-B phosphorylation and *NOS* transcription. PKA activity after (A) injection of NPF2 peptide in G-phase locusts and (B) transcript knockdown of *NPF2* in S-phase locusts (n = 4 replicates, 15–20 locusts/replicate, *P < 0.05, **P < 0.01 with Student’s t-test). (C) p-CREB-B and (D) *NOS* transcription levels after the administration of PKA agonist (Colforsin) in G-phase locusts pre-injected with NPF2 peptide. (E) p-CREB-B and (F) *NOS* transcription levels after the administration of PKA inhibitor (KT5720) in S-phase locusts pre-injected with dsNPF2 (n = 3 replicates, 6–8 locusts/replicate for qPCR, 8–12 locusts/replicate for western blot, one-way ANOVA, *P* < 0.05).

We then investigated whether or not PKA is involved in the regulation of NPF2 on the CREB-B/NOS signaling. The brain PKA activity was down-regulated by NPF2 injection but was promoted by the gene knockdown of *NPF2* (Fig 7A and 7B). Moreover, the administration of PKA agonist (Colforsin) rescued the levels of p-CREB-B and *NOS* transcription, which were reduced by NPF2 injection in gregarious locusts (Fig 7C and 7D). Meanwhile, the injection of PKA inhibitor (KT5720) can fully recover the boosted levels of p-CREB-B and *NOS* transcription induced by *NPF2* knockdown in solitary locusts (Fig 7E and 7F). In summary, these data revealed the essential role of PKA in the transduction of NPF2/CREB-B/NOS signaling involved in locust behavioral phase change.

## Discussion

Our study shows that the transcription factor CREB-B acts as a key mediator of the NPF/NO signaling pathway in regulating phase-related locomotor plasticity in migratory locusts. The essential role of CREB-B in locomotor plasticity is achieved through its direct control of *NOS* transcription under NPF2 regulation, and PKA transmits the effects of NPF2 on p-CREB-B.

By combined using genome walking and dual-luciferase assays, we characterized the promoter sequence of *NOS* gene. The core promoter sequence of *NOS* lies at 150 bp to 120 bp upstream region of the NOS ORF that is required for basic *NOS* transcription. In this region, there might exist other negative regulatory elements because we found that transcriptional activity of *NOS* promoter decreases when more than -140 bp DNA sequence is included in pGL4.1 vector, implying a complex regulatory networks in *NOS* promoter [33]. Apparently, gene transcription is a complex process that not only involves different regulators, but also depends on the sequence characteristics of promoter region [33, 34]. In fact, we bioinformatically predicted multiple CREB binding sites in the promoter region of *NOS*. And all three locust CREB members have active effects in HEK293T cells, although there are some differences in extent probably due to the differential expression levels of three CREB proteins or their distinct transactivation efficiency. In addition, the lower transcription activation effect of CREB3 observed in longer *NOS* promoter might be due to some inhibitory regulators, or more negative regulatory sequence were involved.

Further *in vivo* studies show that only CREB-B can directly regulate *NOS* transcription by interacting with CREB R4, whereas the other two CREBs, *CREB-A* and *CREB3*, do not have significant regulatory effects on *NOS* transcription in the locust brain. CREB-B and its target NOS are extensively expressed in the neurons upon PI which is a brain region that involves in the regulation of locomotor rhythms in insects [35]. The similar localization of CREB-B and NOS provided additional evidence for their interaction and may support their roles in locomotor modulation in the locust. To our best knowledge, this result provides the first evidence showing the regulatory roles of CREB in dynamic *NOS* transcription in insects. A number of studies have reported that CREB family members display diverse biological functions depending on their cell- or tissue- specific distributions in insects. For example, in *Apis mellifera*, CREB is localized in mushroom bodies and is associated with age-dependent labor division [36]. Moreover, CREB has different regulatory effects on the feeding behavior of *Drosophila* when it expresses in neuronal and peripheral tissues [37]. Therefore, three locust CREB proteins may have distinct distributions and functions. Actually, similar phenomena have also been reported in mammal species [38].

Many reports suggested that *NOS* transcription is essential for both neural and behavioral plasticity by controlling NO content under various physiological or external stimuli [39, 40]. The transcriptional regulatory mechanisms of three NOS isoforms in vertebrates such as neural NOS (nNOS, NOS1), inducible NOS (iNOS, NOS2), and epithelial NOS (eNOS, NOS3) have been well studied. *eNOS* is regulated by FOXO in human umbilical vein endothelial cells [41], whereas *iNOS* transcription is controlled by both STAT1 and NF-κB in human fibroblasts [42]. For *nNOS*, multiple TFs have been proposed, such as NF-κB, SP, and ZNF family members [43–45]. To date, only one NOS isoform in insects has been characterized [46]. The phylogenetic analysis suggests that insect NOS and vertebrate NOSs evolve from the same ancestor. It seems that three vertebrate NOSs duplicate from a single gene after evolutionarily divergence with insect NOS (S15 Fig). Although the importance of NO signaling has been reported in many insects [47, 48], the regulatory mechanism underlying NO synthesis is rarely uncovered. This study provides insights into the inducibility of *NOS* expression in response to environmental stimuli in animals.

Our results suggested that the phosphorylation level of CREB-B, but not the transcription level, is involved in the regulation of *NOS* transcription in response to the changes of population density in the locusts. Except for transcription alteration, several kinds of post-translational modifications, including phosphorylation, acetylation, and ubiquitination, have also been reported to control the transcriptional activation of CREB [31, 49, 50]. Phosphorylation, especially at Ser133 in the kinase-inducible domain (KID), is the most conserved indicator for CREB activation in mammals [51]. Our results showed that among the three antibodies that we tested; only the antibody against Ser133 phosphorylation of mammalian CREB1 (homolog for locust CREB-B) can detect the predicted band in the locust brain. We also observed that the phosphorylation level of CREB-B remarkably responded to the population density change. This result is similar to those from other studies; for example, the reduced CREB activity by protracted social isolation in rats [52] and the age-dependent increase in CREB phosphorylation in honeybees [36]. These findings indicate the CREB phosphorylation serves as a common molecular signal in response to the changes in internal or environmental states.

Among the candidate kinases that catalyze the phos-Ser133 of CREB [31, 32], only *PKA* has been demonstrated to be required for p-CREB-B in activating *NOS* transcription in the locust brain in our studies. However, we cannot exclude the potential roles of other several kinases on CREB phosphorylation in other physiological processes or tissues. The PKA-stimulated CREB phosphorylation has been accepted as a critical step for both neural and behavioral plasticity [53, 54]. The importance of PKA has been reported in the behavioral phase transition of desert locusts, *Schistocerca gregaria* [55]. Moreover, PKA has been recognized as a conserved down-stream signal transducer of both dopamine and serotonin [56, 57]. The two neurotransmitters induce behavioral gregarization and solitariness in the migratory locust, respectively [25, 58].

The involvement of PKA in NPF2/CREB-B axis was further validated by a series of pharmacological experiments of manipulating PKA activity. The importance of PKA in NPY-induced behavioral processes has also been revealed in other species. For example, the NPY signaling displays strongly suppressive effects on PKA-sensitized stress response in *Drosophila* [59]. In addition, the cAMP/PKA signal inhibits NPY-induced feeding behavior in rats [58]. We previously reported that the locust NPF2 peptide displays a close evolutionary relationship with NPY [26]. Thus, the NPY/PKA/CREB-B signaling cascade may represent a common mechanism underlying behavioral plasticity in animals. Surprisingly, although cAMP has been well-known as the main upstream messenger for PKA activation [53], we did not find any changes in cAMP level after manipulating either NPF1a or NPF2 peptide when we examined the whole locust brain [26]. One reasonable explanation is that the effects of NPF peptide on cAMP levels might have cell-specific patterns in the locust brain as reported in *Drosophila* [60]. The cAMP level may change only in a few neurons refer to NPF-NO circuit so that we cannot detect its changes in the whole brain. So, further work is needed to validate whether cAMP can link to NPF and PKA in specific regions of the locust brain during phase transition.

Here, we show that NPF2 displays a long-term effect on locomotor activity by negatively regulating p-CREB-B level and subsequent *NOS* transcription in locust brain. Another locust NPF member, NPF1a gene, has been confirmed to be not involved in the regulation of p-CREB-B by the RNAi-induced knockdown, or the injection of either the full length or the truncated peptide. Apparently, p-CREB-B/NOS signal transduction may specifically respond to NPF2 manipulation. According to this work and our previous findings [26], we infer that NPF2, CREB-B, and NOS should have overlap expressions in the same neuron cells of PI in locust brains. The effects of NPF2 on CREB-B/NOS might through autocrine or paracrine manner by disperse from its expressing cells as reported in other species [61, 62].

**Fig 8:**
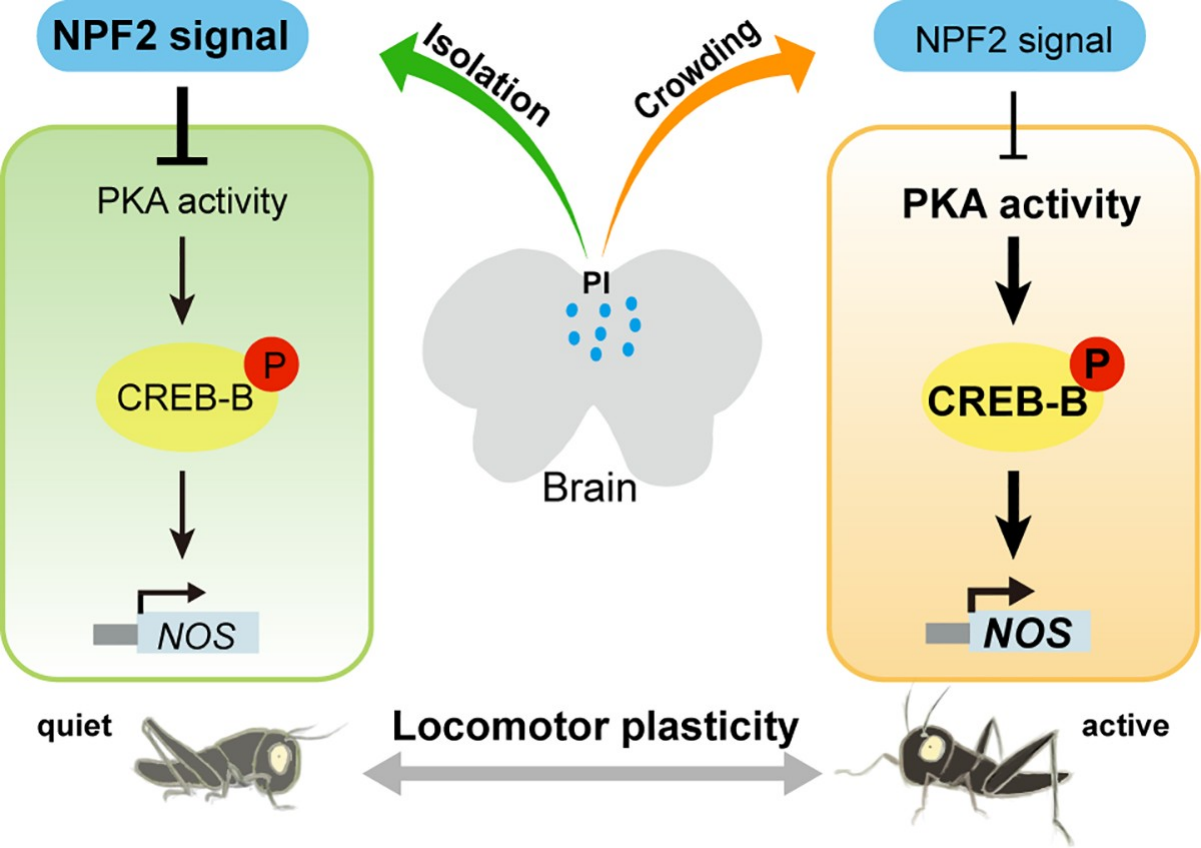
Schematic representation of NPF2/PKA/CREB-B/NOS signaling cascade in the modulation of locomotor plasticity in the locust. During isolation, the up-regulation of NPF2 signal suppresses the *NOS* transcription by inhibiting CREB-B phosphorylation catalyzed by PKA, thus reducing locomotor activity. Upon crowding, the down-regulation of NPF2 signal promotes PKA activity, which in turn activates CREB-B phosphorylation and *NOS* transcription and consequently enhanced locomotor activity. Blue dots in the locust brain indicate NPF2 localization. Bold indicates increased levels or activity.

In summary, this study extends our previous findings by uncovering two key signal components CREB-B and PKA in the NPF2/NO signaling pathway involved in the regulation of phase-related locomotor plasticity (See model in Fig 8). Our findings present a previously undefined regulatory mechanism on *NOS* transcription in insects and shed light on how neuromodulator/TF/effect gene network contributes to environment-induced behavioral plasticity.

## Materials and methods

### Insects and experimental treatments

All locusts were obtained from a colony maintained at the institute of Zoology, Chinese Academy of Sciences. G-phase locusts were reared in large well-ventilated cages (40 cm × 40 cm × 40 cm) under crowded condition (500–1000 insects per cage). S-phase locusts were maintained separately in small boxes (10 cm × 10 cm × 25 cm) under physical, visual, and olfactory isolation from other locusts. Both colonies were maintained at 30 ± 2°C and under 14:10 light/dark photocycle regime. The locusts were fed with fresh wheat seedling and bran [23].

The fourth instar locusts were used for the time-course analysis. For the isolation treatment, the G-phase locusts were separately reared under the solitarious condition as described above, and their brains were collected and snap frozen after 0, 1, 4, and 16 h treatments. For the crowding treatment, two S-phase locusts were reared together with 20 G-phase locusts of the developmental stage in a small cage (10 cm × 10 cm × 10 cm), and their brains were dissected and frozen in liquid nitrogen after 0, 1, 4, and 16 h treatments. Each treatment group consisted of 8–10 locust individuals split approximately between sexes. Four independent biological experiments were conducted for each treatment.

### Peptide injection and drug treatments

Commercially synthesized peptides (BGI, NPF1a truncated peptide: YSQVARPRF-NH2; NPF1a full length peptide (NPF1a-FL): AEAQQADGNKLEGLADALKYLQELDRFYSQVARPRF-NH2; NPF2 peptide: RPERPPMFTSPEELRNYLTQLSDFYASLGRPRF-NH2, 2.5 μg/μl, 2 μl/each locust individual) were injected into the hemolymph in the heads of fourth-instar locusts by using a microinjector. The brains of test locusts were collected 2 h following injections. PKA agonist (Colforsin, 10 μM, 2 μl) was microinjected into the heads of G-phase locusts pretreated with NPF2 peptide, and the locust brains were dissected and frozen 1 h after drug injections. PKA inhibitor (KT5720, 50 μM, 2 μl) was microinjected into the heads of S-phase locusts pre-injected with dsNPF2. Collected brain samples were used for further analysis of p-CREB-B and *NOS* transcription.

### Genome walking and transcription factor prediction

Genome walking experiments were performed by using the Genome Walking Kit (Takara) following the manufacturer’s protocol to obtain the upstream regulatory region of *NOS* gene. Three specific primers (SP1, SP2, and SP3) were designed based on the known upstream sequence of *NOS* (obtained from the locust genome). Three times of thermal asymmetric interlaced PCR were sequentially performed by using the universal primer together with SP1, SP2, and SP3. The PCR product was ligated into the pGEM-Teasy vector (Promega) for sequencing.

The obtained upstream genome sequence of *NOS* was used for the bioinformatic predication of potential *cis*-response elements (CREs). MatInspector program (http://www.genomatix.de/) and TANSFAC program (http://genexplain.com/transfac/) were used for TF analysis to identify the most reliable putative regulatory elements. Candidate TFs raised in both programs were validated in further experiments.

### Construction of vectors

The upstream regions of *NOS* with different lengths were amplified using specific primers and were sub-cloned into the pGL4.1 vector fused with a firefly luciferase (Pp-luc) maker gene (Life Technologies) to validate the predicted regulatory element responding to CREB proteins. The empty pGL4.1 vector was used as a negative control, and the pGL4.1 vector containing SV40 (pGL4.13[luc2/SV40], Promega) promoter sequence that can constitutively transcript was used as a positive control. The Opening reading frame (ORF) sequences of CREB-A, CREB-B, and CREB3 were respectively cloned into pcDNA3.1 expression vector with Flag tag on the C-terminal ends of the target genes.

### Cell culture, transfection, and luciferase report assay

The HEK293T cells were seeded in 500 μl of DMEM medium (Thermo Scientific) in a 24-well plate 1 day before transfection. The pGL4.1-derived constructs (200 ng/well) were separately or co-transfected with the CREB expression vectors (200 ng/well) to the HEK293T cells using Lipofectamine^TM^ 3000 (Invitrogen, California, USA). The pRL-TK that contains a Renilla luciferase (Rr-luc) encoding sequence was co-transfected with the pGL4.1-derived vectors, and was used as an internal control to normalize the transfection efficiency and luciferase activity [63]. All reporter constructs were prepared using the TIANprep MINI Plasmid Kit (TIANGEN, Beijing, China). The cells transfected with different recombinant vectors were cultured for additional 36 h at 37°C for transcriptional activity analysis using the Dual-Glo Luciferase Assay System (Promega) with a luminometer (Promega) according to the manufacturer’s instruction. The luciferase activity was defined as the ratio of Pp-luc activity from pGL4.1-derivative to Rr-luc activity from pRL-TK.

### RNA interference (RNAi)

Double-stranded RNAs (dsRNAs) of target genes were synthesized using T7 RiboMAX Express RNAi system (Premega). dsRNA was firstly microinjected into the brains of the third instar locusts to improve RNAi efficiency and specificity. A second injection was performed at day 1 of the fourth instar locusts (200 ng/locust for each injection). dsGFP was used as the control in all RNAi assays. The brains of test locusts were collected at 48 h following the second injection.

### RNA isolation and qPCR

Total RNA of experimental samples (6–8 locust/sample, four samples were collected for each treatment) was extracted using TRIzol reagent according to the manufacturer’s instruction. RNase-free DNase (1 μl, 1 U/μl, Promega) was added to RNA solution and incubated at 37°C for 30 min to remove genomic DNA, the mixture was then incubated at 65°C for 10 min to inactivate DNase. RNA quantification and reverse transcription were performed as previously described [26]. Gene-specific transcript levels were detected by qPCR using the SYBR Green kit on a LightCycler 480 instrument (Roche). *RP49* was used as the internal reference. The primers are shown in S2 Table.

### Electrophoretic mobility shift assay (EMSA)

Gel-shift assay was conducted using the LightShift Chemiluminescent EMSA Kit (Thermo Scientific, USA) to verify the binding of CREB-B to candidate regulatory regions of NOS promoter. The oligonucleotides were labeled with biotin at the 5′ end and incubated at 95°C for 10 min and then annealed to generate the double-stranded probe by natural cooling. The cold probes (unlabeled probe) or mutant probes were used as competitors of wild type probes. All oligonucleotide probes were synthesized by Invitrogen Company (Shanghai, China).

DNA-binding reactions were conducted in a 20 μl mixture containing 1 μg of nuclear extracts (isolated from the locust brains using Nuclear and Cytoplasmic Protein Extraction Kit, Beyotime), 50 ng of poly (dI-dC), 2.5% glycerol, 0.05% NP-40, 50 mM KCl, 5 mM MgCl_2_, 4 mM EDTA, and 0.25 μM of the biotin-labeled probe at room temperature for 20 min. For the competition assay, cold probes (100 ×) were added to the binding reaction. For the super-shift assay, 2 μg of CREB-B antibody or rabbit IgG was added and incubated for another 30 min at room temperature. The protein-DNA complexes were separated using a 6% polyacrylamide gel and transferred onto nylon membranes. The transferred complex was then exposed to UV light cross-linking for 300 s (254 nm, 1200 mJ). The membrane was incubated with a streptavidin-horseradish peroxidase conjugate and was detected by cemiluminescent nucleic acid detection module (Thermo Scientific).

### Whole-mount immunohistochemistry

Whole-mount immunohistochemistry was performed as described in Hou et al. (2017). Brains of the fourth instar locusts were dissected and fixed in 4% paraformaldehyde (PFA) at 4°C overnight. Polyclonal antibody against p(S133)-CREB1 (Cell signaling, 1:100) and monoclonal mouse antibody against uNOS (Thermo, 1:100) were used as the primary antibodies, which were incubated with locust brains for 48 h at 4°C. Alexa Fluor-488 goat anti-rabbit IgG (1:500; Life Technologies) and Alexa Fluor-568 goat anti-mouse IgG (1:500; Life Technologies) were used as secondary antibody for CREB-B and NOS staining, respectively. Hoechst (1:500) was used for nuclear staining. Fluorescence was examined under the LSM 710 confocal laser-scanning microscope (Zeiss).

### Protein extraction and Western blot

Total proteins from locust brains were extracted using TRIzol reagent following the manufacturer’s instruction. Brain tissues (8–12 insects/sample) were homogenized in 1 ml of TRIzol reagent. After 200 μl of chloroform was added, the aqueous phase was used for total RNA isolation, and the DNA in the phenol phase and interphase was excluded by ethanol precipitation. Proteins were then precipitated by adding isopropyl alcohol for 20 min at room temperature. The protein pellet was washed in a solution containing 0.3 M guanidine hydrochloride in 95% ethanol, followed by 100% ethanol washing. After vacuum drying, the protein pellet was weighed and dissolved in 1% SDS sample buffer to 10 μg/μl.

The protein extracts (100 μg) were electrophoresed on 12% SDS-PAGE gel and then transferred to polyvinylidene difluoride (PVDF) membrane (Millipore). The membrane was incubated with polyclonal antibody against p(S133)-CERB1 (Cell signaling, 1:1000) or monoclonal antibody against H3 (1:5000). Goat anti-rabbit IgG (CoWin, 1:5000) and goat anti-mouse IgG (CoWin, 1:10000) were used as secondary antibody for CREB and H3, respectively. Protein bands were detected by chemiluminescence (ECL kit, Thermo Scientific). The band intensity was analyzed using Quantity One software. Briefly, the targeting bands were selected and the background signal was deducted. Band intensity was presented as the peak area value and normalized by that of nucleoprotein H3.

### Behavioral assay for locomotor activity

Locomotor activity was monitored as previously described [26]. Generally, locust behaviors were detected in a rectangular Perspex arena (40 cm × 30 cm × 10 cm). The locust behaviors were recorded for 6 min by an EthoVision video tracking system. Total distance moved and total duration of movement in the middle 300 s represent the locomotor activity of individual locusts. More than 15 locusts were tested for each experimental treatment according to the sample size used in previous studies [25, 55]. Locusts that did not move in the arena assay were excluded.

### PKA activity assay

PKA activity was examined using the PKA Kinase Assay Kits, Type I (Immunechem) according to the manufacturer’s protocol. The locust brains (15–20 locusts/sample) were collected and homogenized in 200 μl 1 × PBS buffer (0.1 M phosphate buffer, 0.15 M NaCl, pH 7.4). The supernatant (50 μl/well) was added into the substrate plate containing kinase assay dilution buffer. ATP (10 μl/well) was added to initiate the kinase reaction at 30°C for 90 min. After the reaction solution was removed, anti-p-substrate antibodies (40 μl/well) were incubated for 60 min at room temperature. Goat anti-rabbit IgG HRP was used as secondary antibody. TMB solution was used to develop the color indicating reaction activity. OD_450_ was detected to calculate the relative kinase activity. The enzyme activity was normalized to the protein concentration, which was measured by using BCA method.

### Phylogenetic analysis and sequence alignment

The protein sequences of *Drosophila* CREB-A and CREB-B were used to search their homologs in the locust genome and transcriptome database by utilizing the tblastn algorithm. CREB proteins in several representative insects and human were used to construct their phylogenetic relationship by using MEGA software. Neighbor-joining method and Poisson model was used and the number of bootstrap replications was 1000. The sequence identity of three locust CREB proteins was analyzed in GENEDOC software.

### Statistical analysis

All data were statistically analyzed using GraphPad Prism 5 software and presented as mean±s.e.m. Student’s t-test was used for two-group comparison, and one-way ANOVA followed by Turkey’s post-hoc test was used for multi-group comparisons. Differences were considered as statistical significance at *P* < 0.05. All the experiments were performed with at least three independent biological replicates. Numerical data for main figures and supporting figures were shown in S3, S4 and S5 Tables.

## Supporting information

S1 Fig Transcriptional activities of constructs containing the upstream elements of *NOS* with different length. (n = 4 replicates, one-way ANOVA, *P* < 0.05, different letters labeled in columns indicate a significant difference). Numbers indicate the distance from the translation initiation site (+1) of *NOS*. The empty PGL4.1 vector fused with a firefly luciferase (Pp-luc) as the negative control (NC). The pGL4.13[luc2/SV40] vector that can constitutively expression was used as the positive control (PC). The pRL-TK vector that contains a Renilla luciferase (Rr-luc) encoding sequence was used as an internal reference to normalize the cell numbers and the transfection efficiency.
(TIF)

S2 Fig Phylogenetic relationship of CREB proteins in insects and human. Three CREB encoding genes were found in the locust genome and were named as LomCREB-A, LomCREB-B, and LomCREB3 (marked with triangles) according to the phylogenetic analysis. The LomCREB-B protein is close to human CREB1, whereas LomCREB-A and LomCREB3 are evolutionarily divided to the cluster containing human CREB3.
(TIF)

S3 Fig Sequence alignment of three CREB proteins in the locust. LomCREB-A, LomCREB-B, and LomCREB3 share low identity (13.55%).
(TIF)

S4 Fig Over-expression of three CREB proteins in HEK293T cells as validated by Western blot. DNA fragment encoding CREB-A (479 aa), CREB3 (569 aa), or CREB-B (280 aa) followed by a Flag-tag was inserted to pcDNA3.1 expressing vector. Red arrows indicate target proteins. Total protein of cells transiently transfected with pcDNA3.1-CREB-A, pcDNA3.1-CREB3, or pcDNA3.1-CREB-B was used for Western blot analysis. Cells transfected with pcDNA3.1 were used as control. Mouse monoclonal antibody against Flag (CoWin, 1:5000) was used to validate the expression of these proteins.
(TIF)

S5 Fig RNAi efficiency and specificity of three CREB encoding genes validated by qPCR. dsRNA of each gene was microinjected to the brains of 3th instar locusts, and a second injection was performed at day 1 of the fourth instar locusts. RNAi effects were detected at 48 h after the final injection. Data are presented as mean ± s.e.m. (n = 4 replicates, 6–8 locusts/replicate, one-way ANOVA, *P* < 0.05).
(TIF)

S6 Fig EMSA analyzing the binding of the brain nuclear extracts to three predicted CREB-response elements of the *NOS* promoter. (A) EMSA of the nuclear proteins extracted from the brain tissues to CREB R1 of the *NOS* promoter. (B) EMSA of the nuclear proteins extracted from the brain tissues to the CREB R2 of the *NOS* promoter. (C) EMSA of the nuclear proteins extracted from the brain tissues to the CREB R3 of the *NOS* promoter. Nuclear proteins extracted (1 μg) from the locust brain were used to bind with the biotin-labeled probe (CREB R1, CREB R2, and CREB R3). The corresponding cold CREB probes were unlabeled.
(TIF)

S7 Fig LomCREB-B and HosCREB1 share conserved phosphorylation site in their kinase-inducible (KID) domains. (A) Sequence alignment of the KID domain of LomCREB-B (87–130 aa) and HosCREB1 (108–153 aa). (B), (C), and (D) Validation of LomCREB-B phosphorylation using antibodies against specific phosphorylation sites of HosCREB1. The LomCREB-B was phosphorylated at Ser110 detected by anti-p(S133)-CREB1. (D) Validation of LomCREB-B phosphorylation by transcription knockdown experiment. The western band recognized by anti-p(S133)-CREB1 was significantly decreased by *LomCREB-B* gene knockdown, and Histone 3 (H3) was used as the inner reference.
(TIF)

S8 Fig Subcellular localization of CREB-B in the pars intercerebralis (PI) of the locust brain. CREB-B mainly localized in the nuclei. Polyclonal antibody against p(S133)-CREB1 (1:100) were used in the immunohistochemistry assay. Green indicates CREB-B staining, whereas blue indicates nuclei staining. Bar represents 100 μm.
(TIF)

S9 Fig Total distance moved (TDM) and total duration of movement (TDMV) in S-phase locusts after transcript knockdown of *CREB-B* (n ≥ 15 locusts, Student’s t-test). n.s. indicates not significant. (TIF)

S10 Fig **Time course of p-CREB-B levels during the (A) the isolation of G-phase locusts and (B) the crowding of S-phase locusts** (n = 3 replicates, 8–12 locusts/replicate)
(TIF)

S11 Fig **(A) Effects on p-CREB-B level after injection NPF2 peptide in G-phase locusts. (B) Effects on p-CREB-B level after transcript knockdown of *NPF2* in S-phase locusts** (n = 4 replicates, 8–12 locusts/ replicate).
(TIF)

S12 Fig Effects on p-CREB-B level after injection full length NPF1a peptide (NPF1a-FL) in G-phase locusts. (A) Western blot detected by antibody against p-CREB-B. (B) Statistical data for band intensity of (A) (n = 3 replicates, 8–12 locusts/replicate).
(TIF)

S13 Fig mRNA level of *CREB-B* in S-phase locusts after knockdown of *NPF2* gene in crowded (16 h) S-phase locusts (n = 4 replicates, 6–8 locusts/replicate). (TIF)

S14 Fig Effects on CREB-B phosphorylation and *NOS* transcription levels after knockdown of candidate kinases. (A) RNAi efficiency of the gene knockdown of *AKT*, *CAMK4*, *CAMK2*, *PKC*, and *S6K*, respectively (n = 3 replicates, 6–8 locusts/replicate, Student’s *t*-test, *P < 0.05; **P < 0.01). (B) CREB-B phosphorylation level after gene knockdown of *AKT*, *CAMK4*, *CAMK2*, *PKC*, and *S6K*, respectively (n = 3 replicates, 8–12 locusts/replicate). (C) *NOS* transcription level after the gene knockdown of *AKT*, *CAMK4*, *CAMK2*, *PKC*, and *S6K* (n = 3 replicates, 6–8 locusts/replicate).
(TIF)

S15 Fig Phylogenetic relationship of NOS proteins in insects and vertebrates. The insect NOS proteins are evolutionally divergent from all three NOS isoforms from vertebrates, including Xenopus, Mouse, Gorilla, and Human.
(TIF)

S1 Table Candidate kinases that can catalyze CREB-B phosphorylation at serine 110 site predicted by NetPhos 3.1 program. (XLSX)

S2 Table Primers used in q-PCR and RNAi expriments. Red font indicates T7 promoter sequence.
(XLSX)

S3 Table Raw data for the luciferase assay in Fig 1 and S1 Fig. (XLSX)

S4 Table Numerical data for main figures. (XLSX)

S5 Table Numerical data for supporting figures. (XLSX)
