# Supplementary figures and images for: CREB-B acts as a key mediator of NPF/NO pathway involved in phase-related locomotor plasticity in locusts
Source: PLoS Genet. 2019 May 31;15(5):e1008176. doi: 10.1371/journal.pgen.1008176 (PMC6561586; doi:10.1371/journal.pgen.1008176)

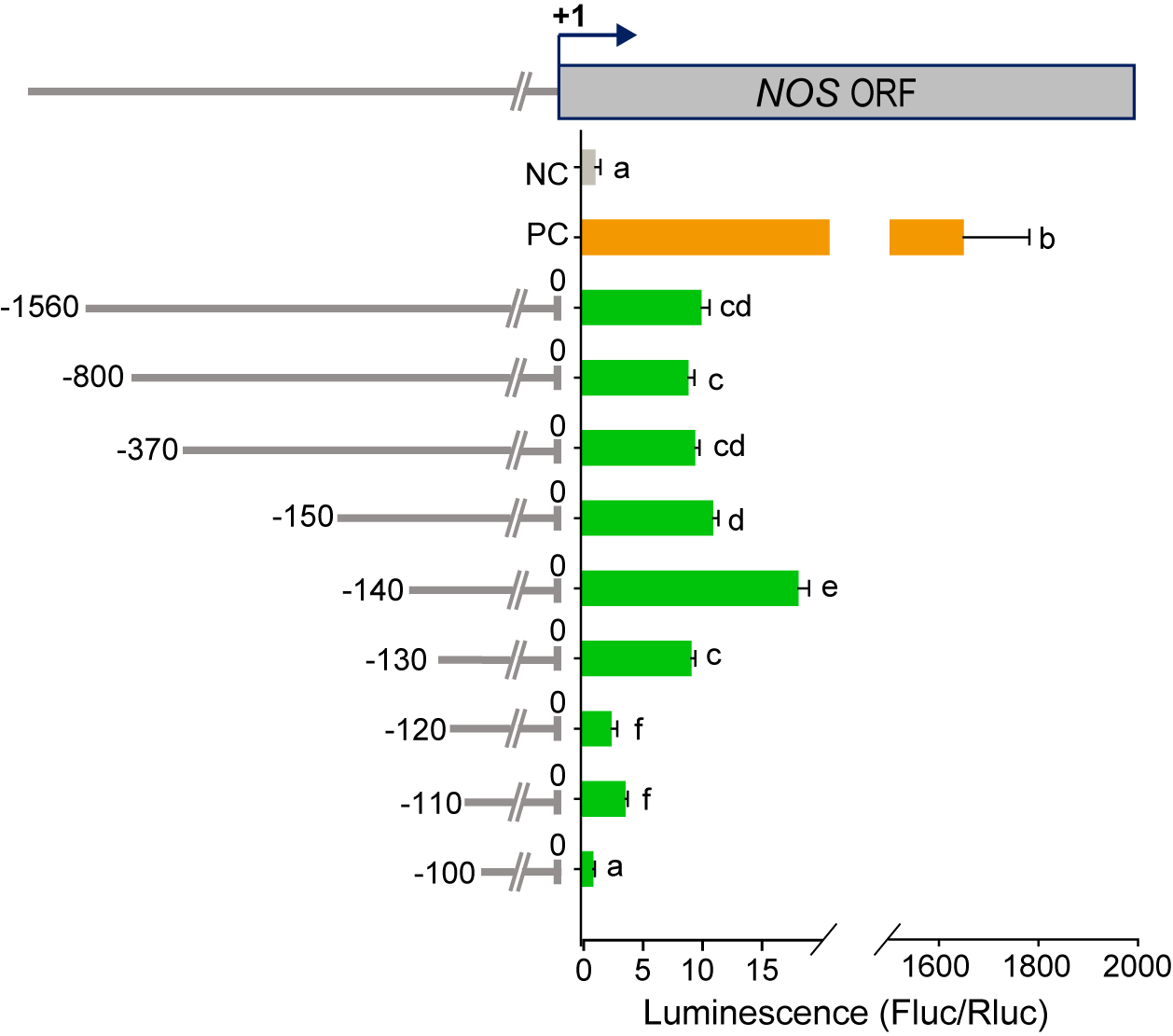

Supplement: S1 Fig — (n = 4 replicates, one-way ANOVA, P < 0.05, different letters labeled in columns indicate a significant difference). Numbers indicate the distance from the translation initiation site (+1) of NOS. The empty PGL4.1 vector fused with a firefly luciferase (Pp-luc) as the negative control (NC). The pGL4.13[luc2/SV40] vector that can constitutively expression was used as the positive control (PC). The pRL-TK vector that contains a Renilla luciferase (Rr-luc) encoding sequence was used as an internal reference to normalize the cell numbers and the transfection efficiency. (TIF) [file pgen.1008176.s001.tif]

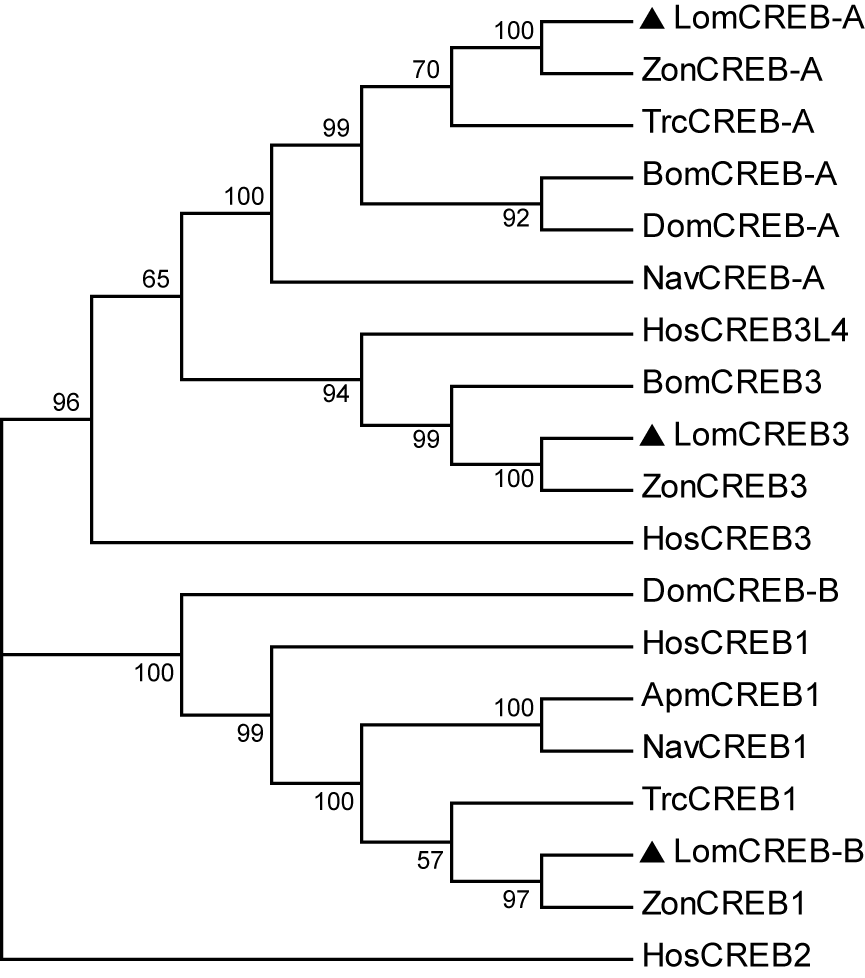

Supplement: S2 Fig — Three CREB encoding genes were found in the locust genome and were named as LomCREB-A, LomCREB-B, and LomCREB3 (marked with triangles) according to the phylogenetic analysis. The LomCREB-B protein is close to human CREB1, whereas LomCREB-A and LomCREB3 are evolutionarily divided to the cluster containing human CREB3. (TIF) [file pgen.1008176.s002.tif]

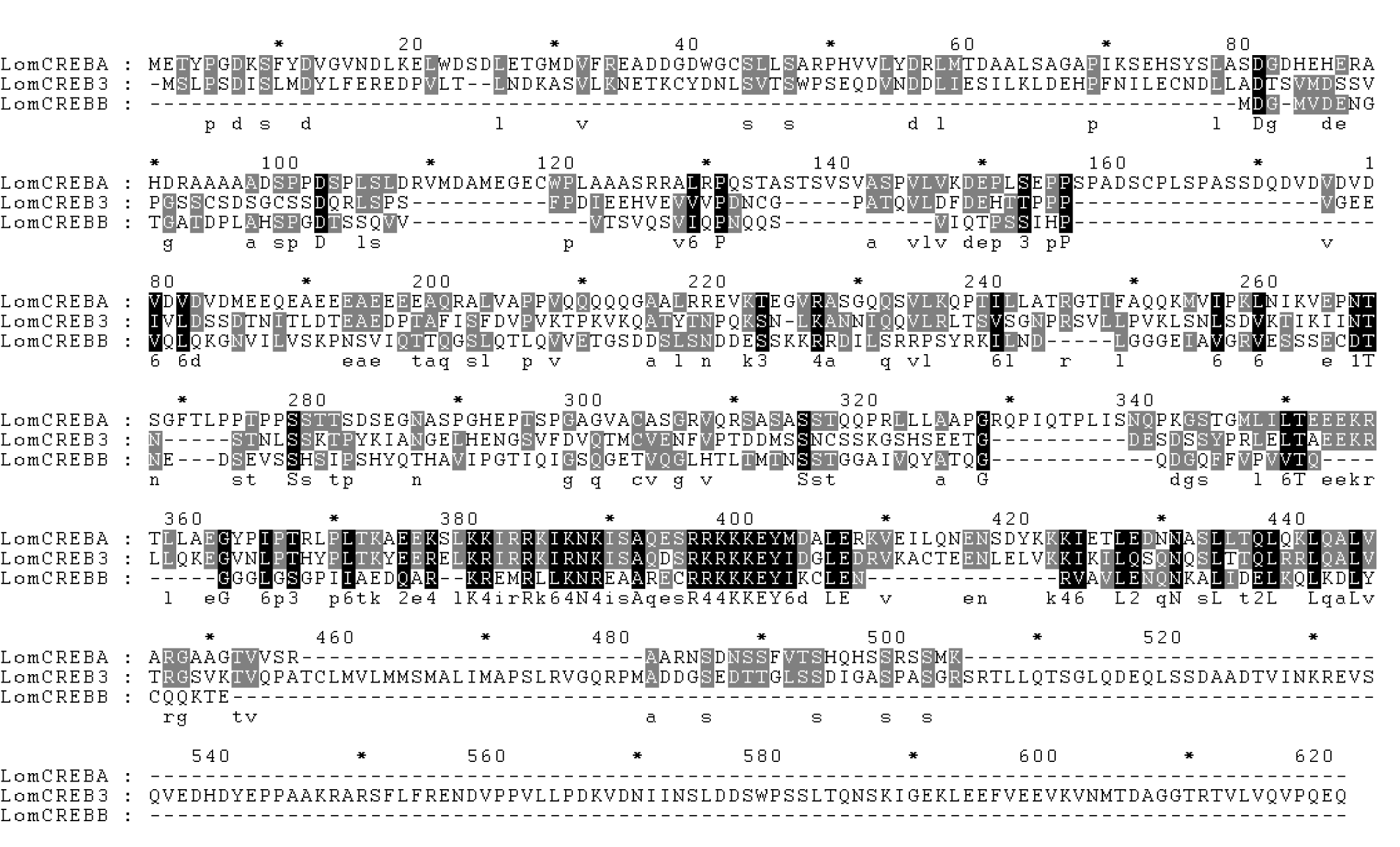

Supplement: S3 Fig — LomCREB-A, LomCREB-B, and LomCREB3 share low identity (13.55%). (TIF) [file pgen.1008176.s003.tif]

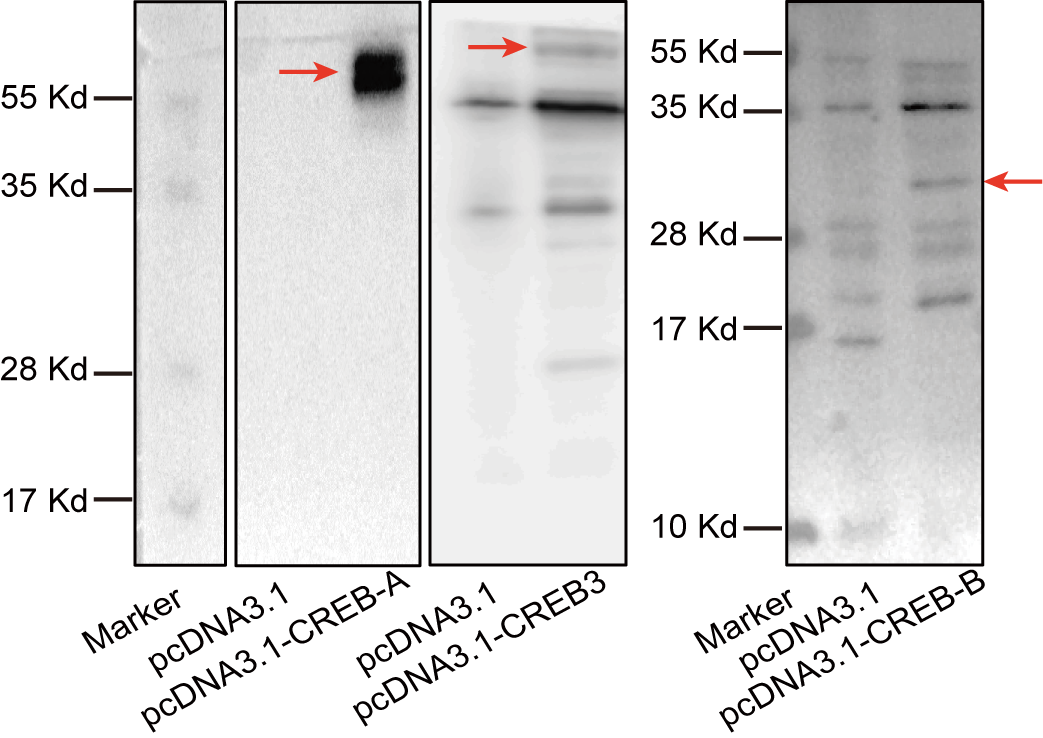

Supplement: S4 Fig — DNA fragment encoding CREB-A (479 aa), CREB3 (569 aa), or CREB-B (280 aa) followed by a Flag-tag was inserted to pcDNA3.1 expressing vector. Red arrows indicate target proteins. Total protein of cells transiently transfected with pcDNA3.1-CREB-A, pcDNA3.1-CREB3, or pcDNA3.1-CREB-B was used for Western blot analysis. Cells transfected with pcDNA3.1 were used as control. Mouse monoclonal antibody against Flag (CoWin, 1:5000) was used to validate the expression of these proteins. (TIF) [file pgen.1008176.s004.tif]

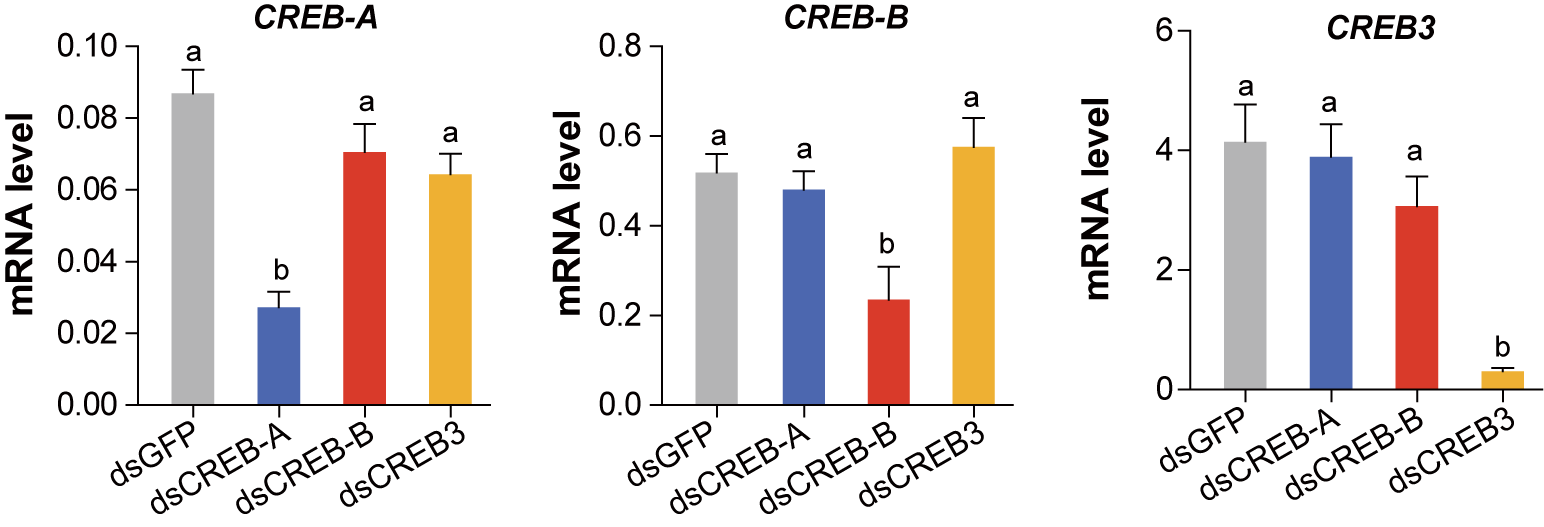

Supplement: S5 Fig — dsRNA of each gene was microinjected to the brains of 3th instar locusts, and a second injection was performed at day 1 of the fourth instar locusts. RNAi effects were detected at 48 h after the final injection. Data are presented as mean ± s.e.m. (n = 4 replicates, 6–8 locusts/replicate, one-way ANOVA, P < 0.05). (TIF) [file pgen.1008176.s005.tif]

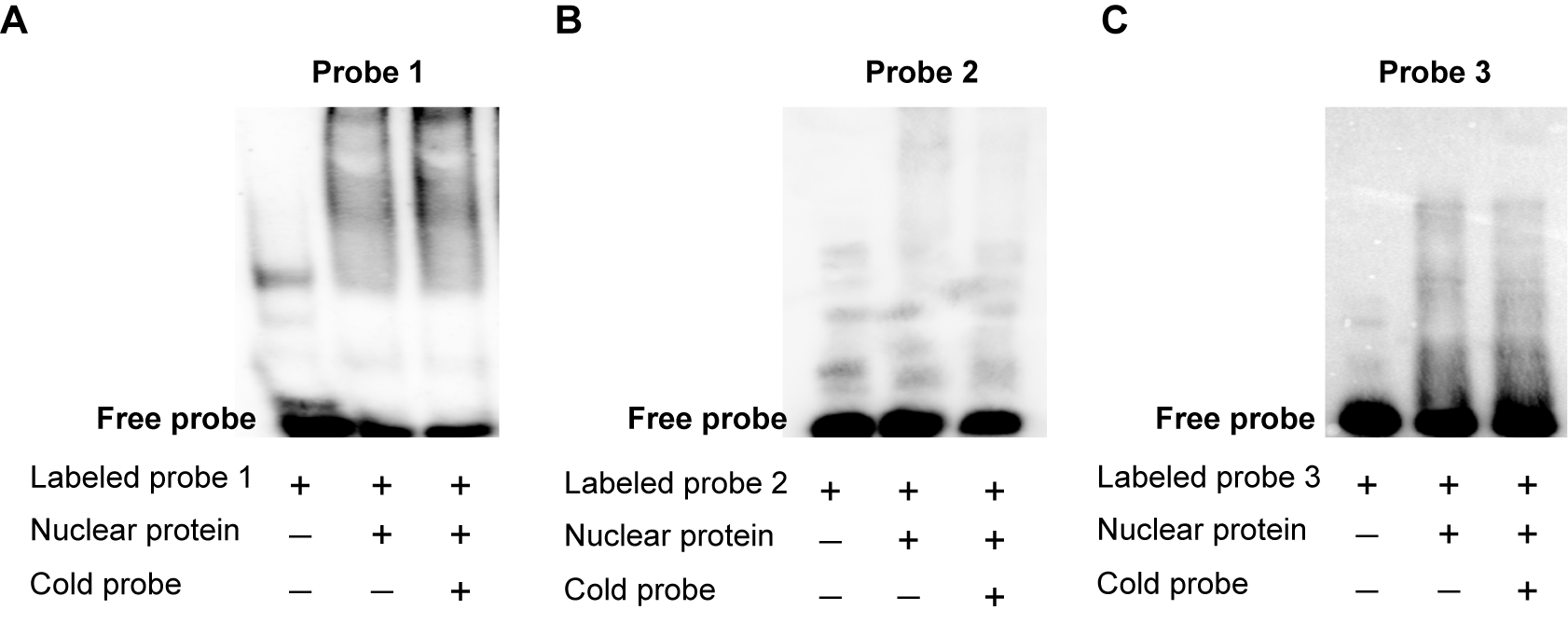

Supplement: S6 Fig — (A) EMSA of the nuclear proteins extracted from the brain tissues to CREB R1 of the NOS promoter. (B) EMSA of the nuclear proteins extracted from the brain tissues to the CREB R2 of the NOS promoter. (C) EMSA of the nuclear proteins extracted from the brain tissues to the CREB R3 of the NOS promoter. Nuclear proteins extracted (1 μg) from the locust brain were used to bind with the biotin-labeled probe (CREB R1, CREB R2, and CREB R3). The corresponding cold CREB probes were unlabeled. (TIF) [file pgen.1008176.s006.tif]

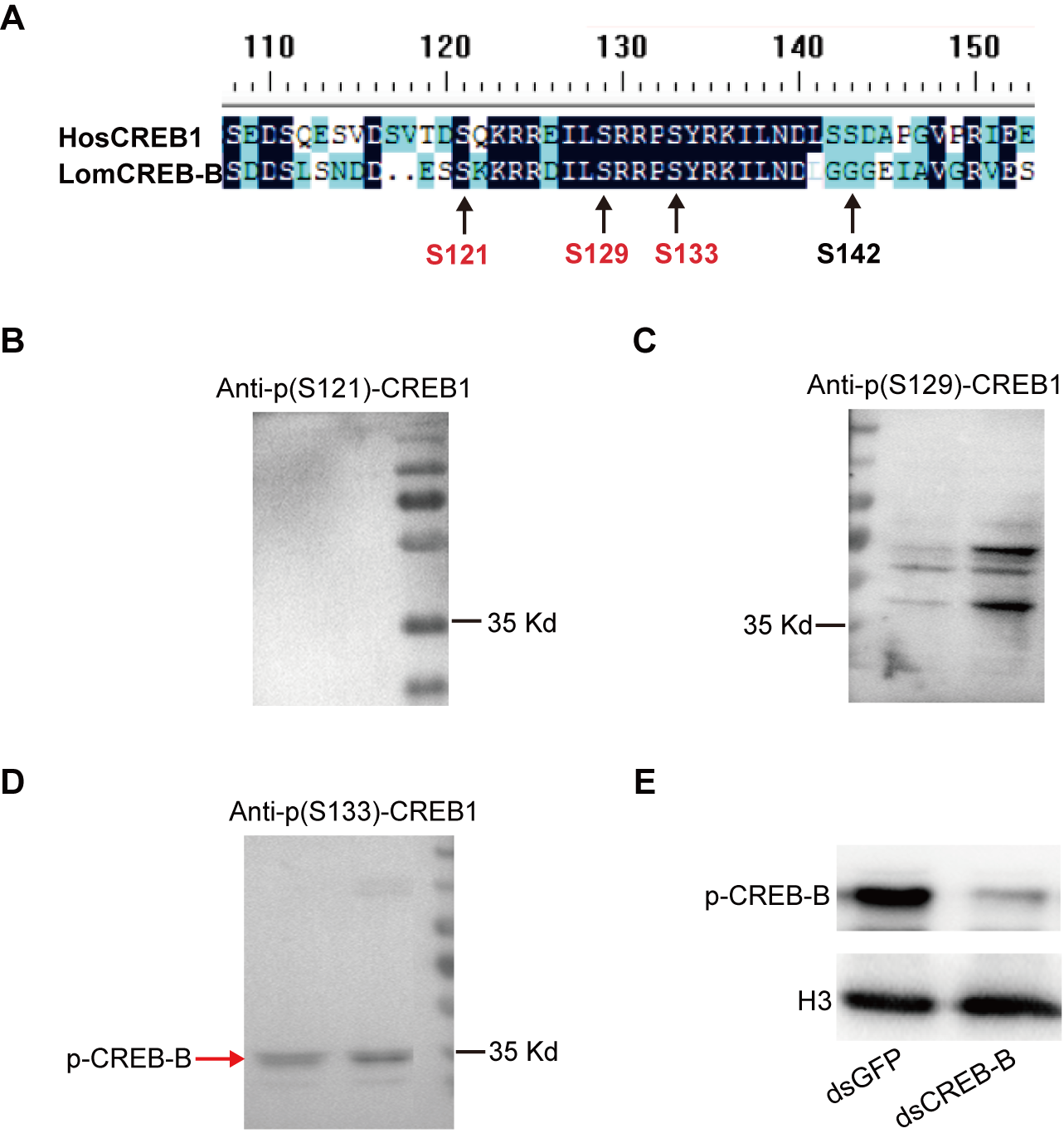

Supplement: S7 Fig — (A) Sequence alignment of the KID domain of LomCREB-B (87–130 aa) and HosCREB1 (108–153 aa). (B), (C), and (D) Validation of LomCREB-B phosphorylation using antibodies against specific phosphorylation sites of HosCREB1. The LomCREB-B was phosphorylated at Ser110 detected by anti-p(S133)-CREB1. (D) Validation of LomCREB-B phosphorylation by transcription knockdown experiment. The western band recognized by anti-p(S133)-CREB1 was significantly decreased by LomCREB-B gene knockdown, and Histone 3 (H3) was used as the inner reference. (TIF) [file pgen.1008176.s007.tif]

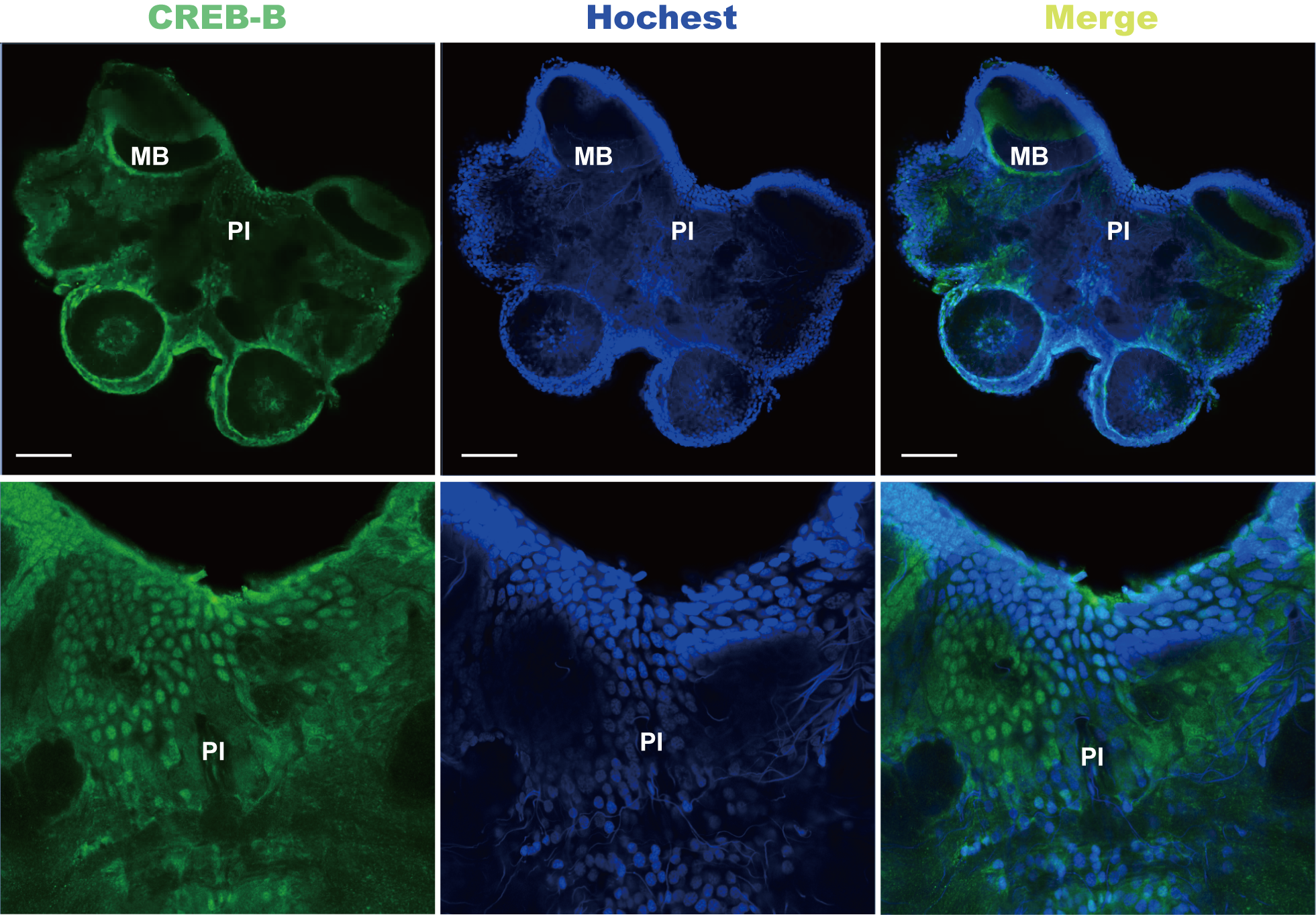

Supplement: S8 Fig — CREB-B mainly localized in the nuclei. Polyclonal antibody against p(S133)-CREB1 (1:100) were used in the immunohistochemistry assay. Green indicates CREB-B staining, whereas blue indicates nuclei staining. Bar represents 100 μm. (TIF) [file pgen.1008176.s008.tif]

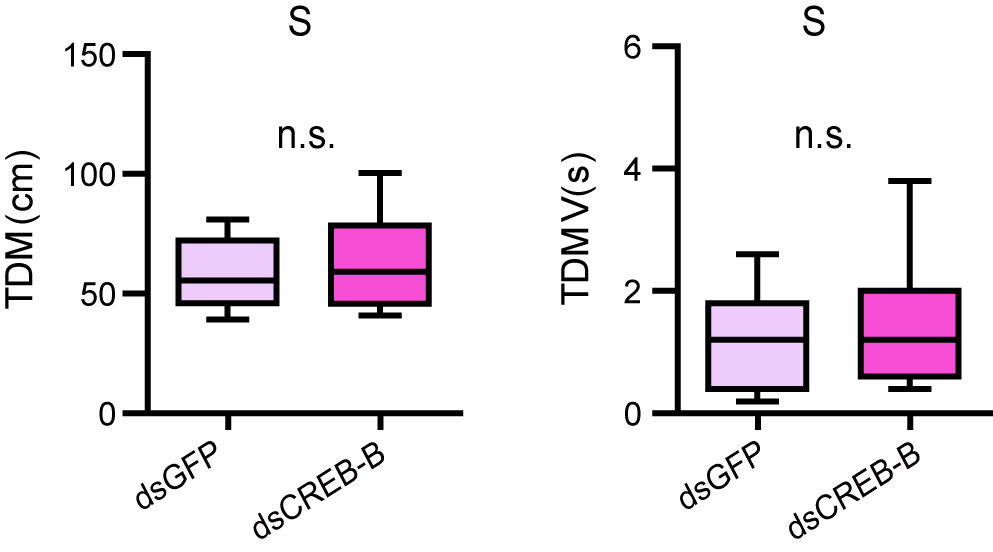

Supplement: S9 Fig — (TIF) [file pgen.1008176.s009.tif]

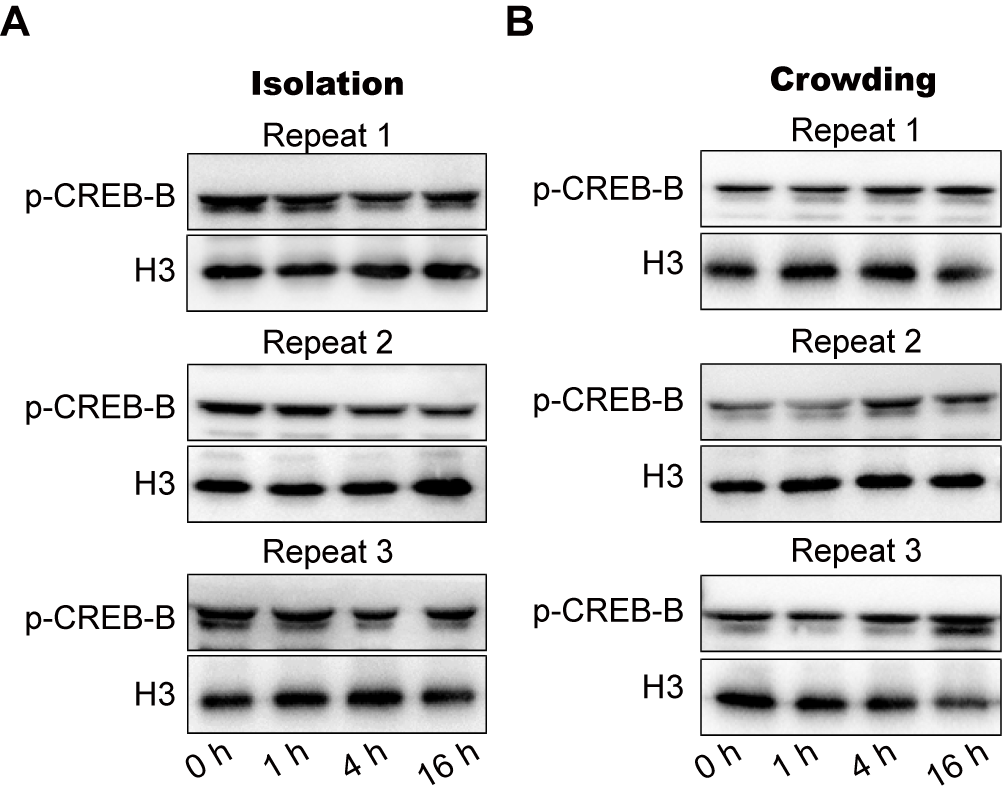

Supplement: S10 Fig — Time course of p-CREB-B levels during the (A) the isolation of G-phase locusts and (B) the crowding of S-phase locusts (n = 3 replicates, 8–12 locusts/replicate) (TIF) [file pgen.1008176.s010.tif]

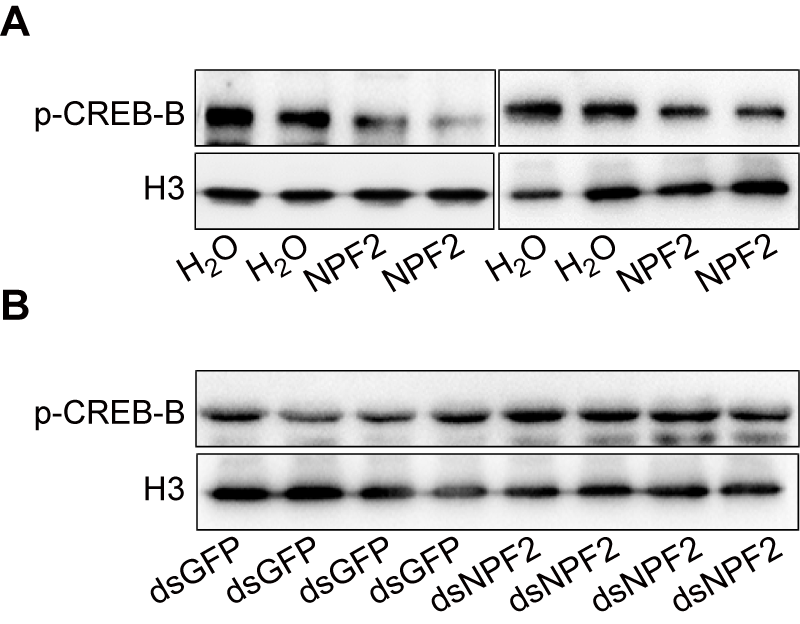

Supplement: S11 Fig — (A) Effects on p-CREB-B level after injection NPF2 peptide in G-phase locusts. (B) Effects on p-CREB-B level after transcript knockdown of NPF2 in S-phase locusts (n = 4 replicates, 8–12 locusts/ replicate). (TIF) [file pgen.1008176.s011.tif]

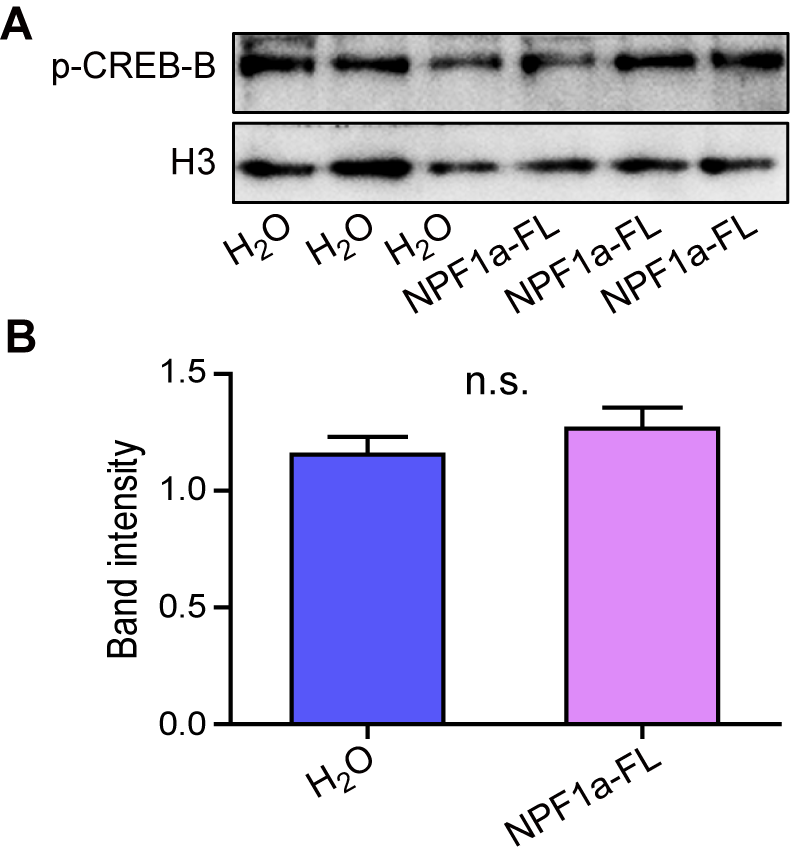

Supplement: S12 Fig — (A) Western blot detected by antibody against p-CREB-B. (B) Statistical data for band intensity of (A) (n = 3 replicates, 8–12 locusts/replicate). (TIF) [file pgen.1008176.s012.tif]

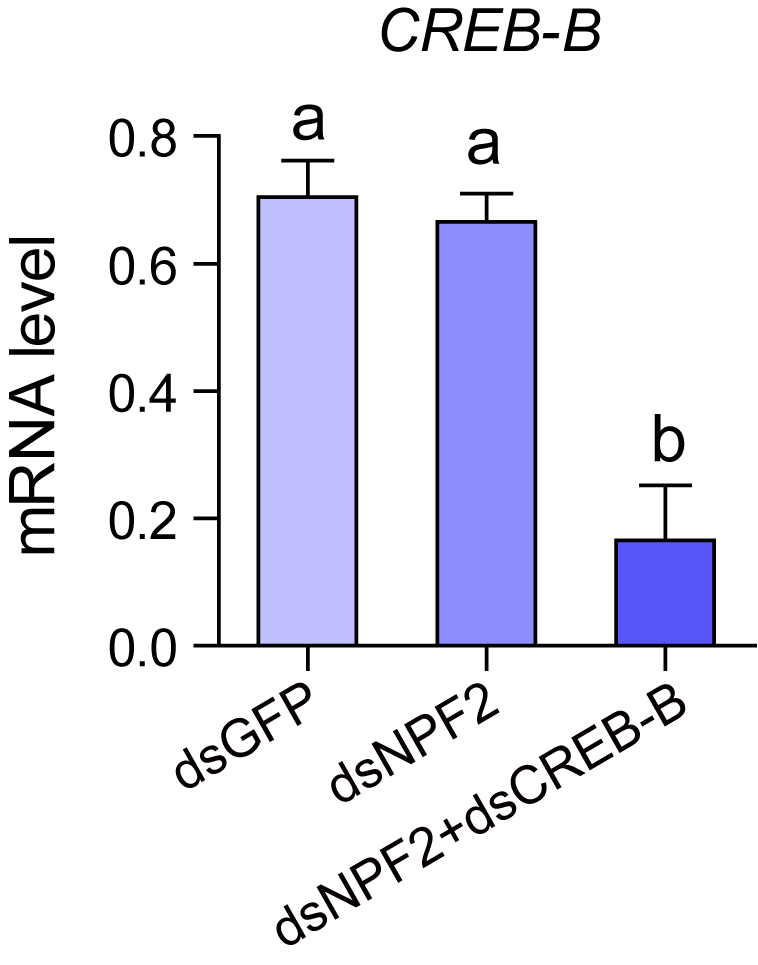

Supplement: S13 Fig — (TIF) [file pgen.1008176.s013.tif]

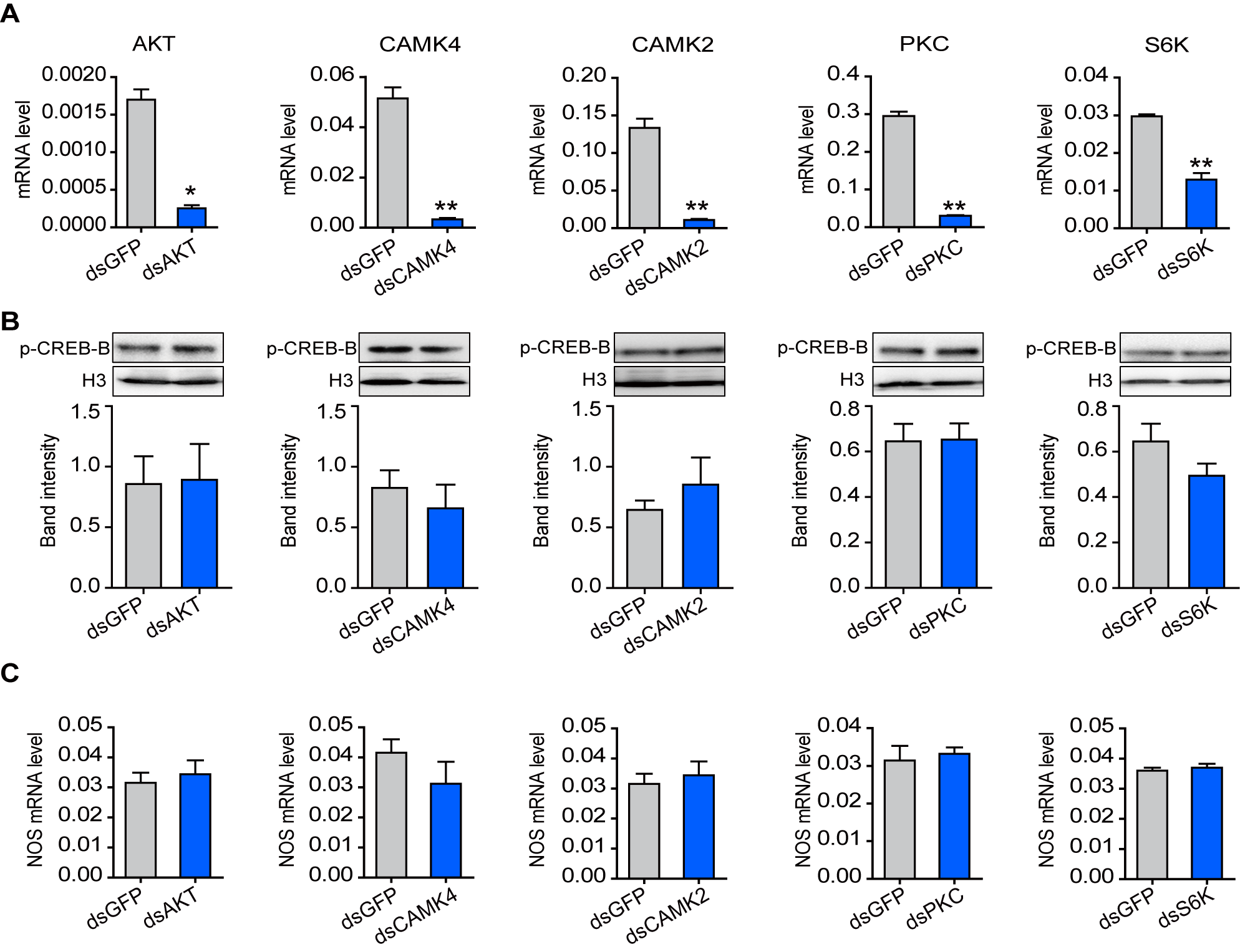

Supplement: S14 Fig — (A) RNAi efficiency of the gene knockdown of AKT, CAMK4, CAMK2, PKC, and S6K, respectively (n = 3 replicates, 6–8 locusts/replicate, Student’s t-test, *P < 0.05; **P < 0.01). (B) CREB-B phosphorylation level after gene knockdown of AKT, CAMK4, CAMK2, PKC, and S6K, respectively (n = 3 replicates, 8–12 locusts/replicate). (C) NOS transcription level after the gene knockdown of AKT, CAMK4, CAMK2, PKC, and S6K (n = 3 replicates, 6–8 locusts/replicate). (TIF) [file pgen.1008176.s014.tif]

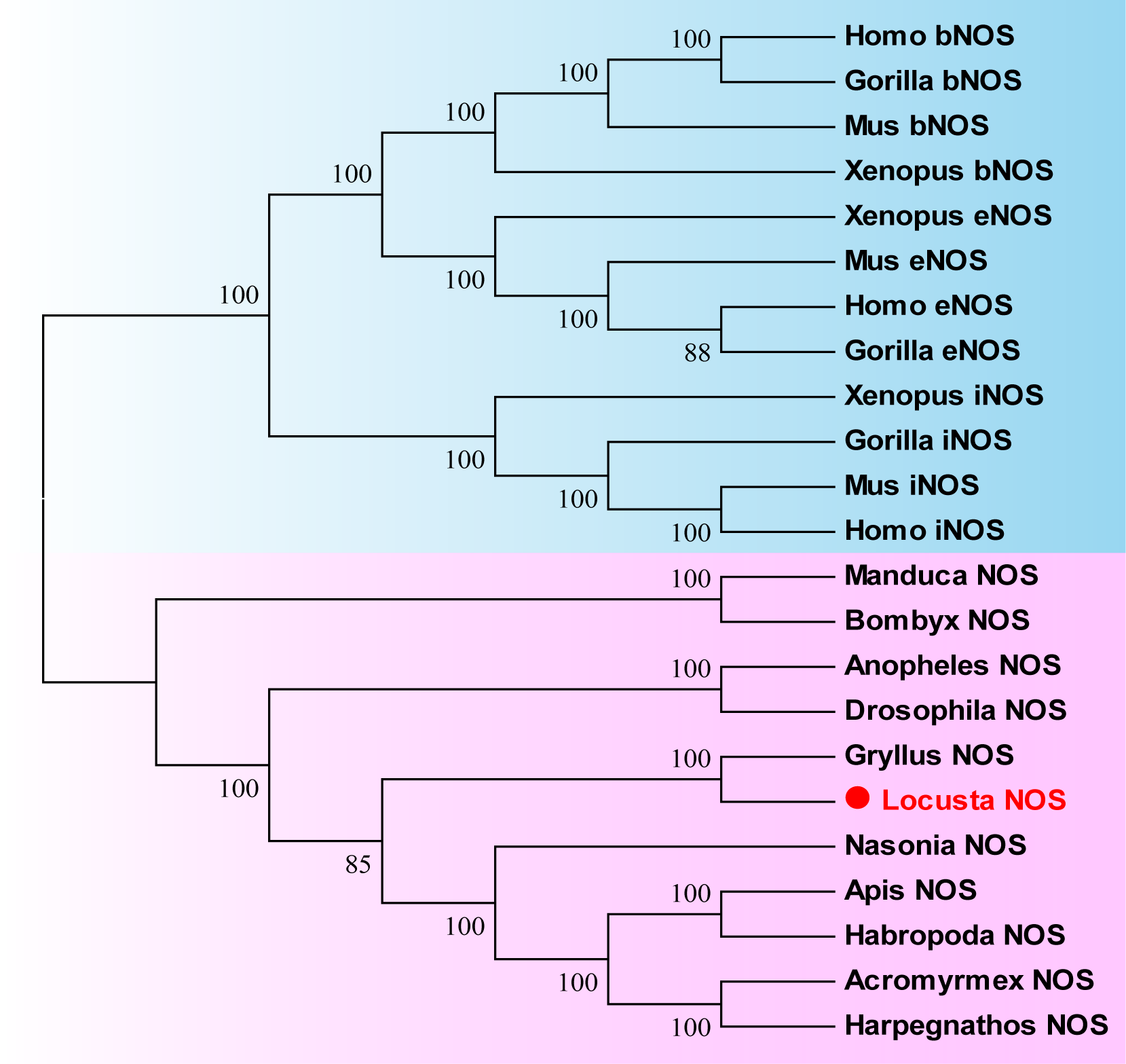

Supplement: S15 Fig — The insect NOS proteins are evolutionally divergent from all three NOS isoforms from vertebrates, including Xenopus, Mouse, Gorilla, and Human. (TIF) [file pgen.1008176.s015.tif]
